# Equivalent Electrical Circuit Representations of AC Quantized Hall Resistance Standards

**DOI:** 10.6028/jres.104.033

**Published:** 1999-12-01

**Authors:** M. E. Cage, A. Jeffery, J. Matthews

**Affiliations:** National Institute of Standards and Technology, Gaithersburg, MD 20899-0001; University of Maryland, College Park, MD 20742-4111

**Keywords:** ac quantum Hall effect, equivalent electrical circuits, intrinsic impedance standard, multi-series connections, quantized Hall resistance, parasitic impedances

## Abstract

We use equivalent electrical circuits to analyze the effects of large parasitic impedances existing in all sample probes on four-terminal-pair measurements of the ac quantized Hall resistance *R*_H_. The circuit components include the externally measurable parasitic capacitances, inductances, lead resistances, and leakage resistances of ac quantized Hall resistance standards, as well as components that represent the electrical characteristics of the quantum Hall effect device (QHE). Two kinds of electrical circuit connections to the QHE are described and considered: single-series “offset” and quadruple-series. (We eliminated other connections in earlier analyses because they did not provide the desired accuracy with all sample probe leads attached at the device.) Exact, but complicated, algebraic equations are derived for the currents and measured quantized Hall voltages for these two circuits. Only the quadruple-series connection circuit meets our desired goal of measuring *R*_H_ for both ac and dc currents with a one-standard-deviation uncertainty of 10^−8^ *R*_H_ or less during the same cool-down with all leads attached at the device. The single-series “offset” connection circuit meets our other desired goal of also measuring the longitudinal resistance *R_x_* for both ac and dc currents during that same cool-down. We will use these predictions to apply small measurable corrections, and uncertainties of the corrections, to ac measurements of *R*_H_ in order to realize an intrinsic ac quantized Hall resistance standard of 10^−8^ *R*_H_ uncertainty or less.

## 1. Introduction

The integer quantum Hall effect (QHE) [1–3] is being used in many laboratories [4–9] in an attempt to realize an intrinsic ac resistance standard by employing ac bridges to compare ac quantized Hall resistances *R*_H_ with ac reference standards. Unfortunately, the measured values of the ac quantized Hall resistances *R*_H_ have varied with the applied frequency *f* of the current, and differed from the dc value of *R*_H_ by at least the factor 10^−7^ *R*_H_ at a frequency *f* of 1592 Hz (where the angular frequency *ω* = 2π*f* is 10^4^ rad/s). Furthermore, sample probe leads had to be removed at the device in these experiments to even achieve this disappointing result. Removing sample probe leads at the device precludes measuring many of the parasitic impedances, which we will show here can be important. Removing leads also precludes measuring both *R*_H_ and the longitudinal resistance *R_x_* during the same cool-down, which has been found to be necessary [10] in order to obtain reliable values of *R*_H_ with direct (dc) currents, and may be even more vital in ac measurements because the measured ac values of *R_x_* have also been found to be frequency-dependent [4,5,11].

Our desired ultimate goal at NIST is to measure both *R*_H_ and the longitudinal resistance *R_x_* during the same cool-down using both ac and dc currents with all sample probe leads attached at the device, and to do this with a one-standard-deviation uncertainty of 10^−8^ *R*_H_ or less. We require an uncertainty this small because the combined one-standard deviation uncertainty of the entire NIST calculable capacitor chain [12] which provides the System International (SI) value of *R*_H_ is only 2.4 × 10^−8^ *R*_H_. Thus, we need to do better than that combined uncertainty in order to verify and replace parts of the calculable capacitor chain.

The observed 10^−7^ *R*_H_ deviation due to the frequency dependence of *R*_H_ in ac quantized Hall resistance experiments is therefore a serious problem that must be addressed. This is the final paper in a series that tries to adequately reduce or account for the frequency-dependent effects of parasitic impedances existing in all ac quantized Hall resistance standards. We believe this paper accomplishes that purpose, and that an intrinsic ac quantized Hall resistance standard can be realized with an uncertainty of 10^−8^ *R*_H_ or less with all sample probe leads attached.

## 2. Our Strategy

We investigate the effects of parasitic impedances on measurements of the ac quantized Hall resistance *R*_H_ by using equivalent electrical circuits to represent ac quantized Hall resistance standards. Each circuit component is a discrete, externally measurable quantity. The discrete components of the ac quantized Hall resistance standards consist of: (a) capacitances and leakage resistances to the shields of the sample probe; (b) series inductances and series resistances of the sample probe leads; (c) wire-to-wire capacitances between pairs of conducting surfaces of the quantum Hall effect device, the sample holder, and the bonding wires between them; and (d) quantized Hall resistances, longitudinal resistances, and voltage generators within the quantum Hall effect devices themselves. These circuit components account for the externally measurable device parameters and parasitic impedances of the standards. We believe these equivalent circuits are the simplest complete representations of ac quantized Hall resistance standards.

Exact algebraic equations for the currents and quantum Hall effect voltages of the standards are derived from these circuits. The equations are complicated; it required many months to derive and check the algebra, and to numerically confirm test cases for each of the two circuits finally considered. We were therefore very selective in choosing which circuits to analyze.

Considerable effort had been expended in our previous papers to reach this final selection and analysis stage. Reference [13] calculated the effects of longitudinal resistances in quantum Hall effect devices for various kinds of multiple-connections to the devices. (Multiple-connections will be explained in Sec. 7.) The exact equations for the currents and quantum Hall voltages derived in Ref. [13] should be used for the corrections that result from parasitic resistances in the sample probes when making dc measurements of quantized Hall resistance standards. Reference [14] described precision experimental verifications of these corrections for several multiply-connected dc circuits. Reference [15] found approximate equations for some of the currents in single-series “normal”, double-series, and quadruple-series connection circuits when the effects of capacitances-to-shield and leakage resistances within the sample probes were included. Most of the capacitances-to-shield arise from the capacitances between the inner and outer conductors of the coaxial leads and connectors within the ac quantized Hall resistance standard; a smaller amount arises from the capacitances between the quantum Hall effect device plus sample holder and the surrounding conducting surfaces of the sample probe. Finally, Ref. [16] found the exact equations for the effects of capacitances-to-shield, series resistances, series inductances, and leakage resistances within the sample probes; this analysis was done for single-series “normal”, single-series “offset”, double-series, and quadruple-series connections.

Two circuits in Ref. [16] showed promise for further consideration: single-series “offset” and quadruple-series connections to the quantum Hall effect device. In this paper, we now include in these two circuits the effects of wire-to-wire capacitances between pairs of inner conductors. Significant wire-to-wire capacitances can exist between pairs of conducting surfaces of the quantum Hall effect device, the sample holder, and the bonding wires between them.

A brief explanation of the dc quantum Hall effect is given in Sec. 3. Section 4 describes our equivalent electrical circuit model of a quantum Hall effect device, and Sec. 5 incorporates that circuit into the equivalent circuit of an ac quantized Hall resistance standard. The single-series “offset” circuit is explained and analyzed in Sec. 6. We will find that the single-series “offset” circuit does *not* meet our goal of measuring the quantized Hall resistance *R*_H_ to an uncertainty of 10^−8^ *R*_H_ or less, but it *does* provide the means to adequately measure the ac longitudinal resistance *R_x_* and to then apply corrections to the measurement. Section 7 considers the quadruple-series connection circuit; the predictions for that circuit *do* meet our measurement uncertainty goal for *R*_H_.

## 3. DC Quantum Hall Effect

The quantum Hall effect (QHE) has been successfully used as an intrinsic dc resistance standard. In the integer dc QHE [1–3], the Hall resistance *R*_H_ of the *i*th plateau of a fully-quantized, two-dimensional electron gas (2DEG) is *R*_H_*(i)* = *V*_H_(*i*)/*I*_T_, where *V*_H_(*i*) is the quantum Hall voltage measured between potential probes located on opposite sides of the device, and *I*_T_ is the total current flowing between the source and drain current contacts at the ends of the device. Under ideal conditions, the values of *R*_H_*(i)* in standards-quality devices satisfy the relationships *R*_H_*(i)* = *h*/(*e*^2^*i*) = *R*_K_/*i*, where *h* is the Planck constant, *e* is the elementary charge, *i* is an integer, and *R*_K_ is the von Klitzing constant, *R*_K_≈ 25 812.807 Ω [17]. However, the conditions are not always ideal. The values of *R*_H_can vary with the device temperature *T* [18] and with the applied current *I*_T_ [19]. Thus the measured dc values of *R*_H_*(i)* are not necessarily equal to *h*/(*e*^2^*i*).

The current flow within the 2DEG is nearly dissipationless in the quantum Hall plateau regions of high-quality devices, and the longitudinal resistances *R_x_*(*i*) of this standard become very small over ranges of magnetic field in which quantized Hall resistance plateaus are observed. The dc longitudinal resistance is defined to be *R_x_*(*i*) = *V_x_*(*i*)/*I*_T_, where *V_x_*(*i*) is the measured longitudinal voltage drop between potential probes located on the same side of the device. The dc values of *R_x_*(*i*) can also be temperature [18] and current [19] dependent.

## 4. Equivalent Electrical Circuit of a QHE Device

**Fig. 1:**
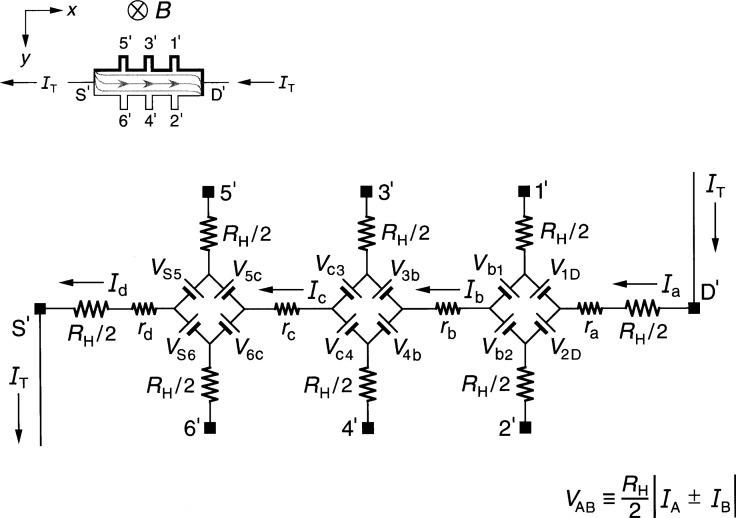
An equivalent electrical circuit, using diamond-shaped arrays of internal voltage generators *V*_AB_, of a quantum Hall effect device when the device is operated on a quantized Hall resistance plateau and has finite longitudinal resistance. The symbols and figure inset are explained in Sec. 4.

The inset of Fig. 1 shows a QHE device for the case when: (a) the magnetic flux density *B* is directed into the figure from above; and (b) an applied current *I*_T_ of *negatively-charged electrons* enters the device source contact pad S*′* and exits the drain contact pad D*′*. Under these conditions the drain contact pad D*′* and the potential probe contact pads 1*′*, 3*′*, and 5*′* at the device periphery are at higher potentials than contact pads S*′*, 2*′*, 4*′*, and 6*′*. If a ground potential is applied near the source S*′*, then contacts D*′*, 1*′*, 3*′*, and 5*′* have potentials near the quantum Hall voltage *V*_H_(*i*), and contacts S*′*, 2*′*, 4*′*, and 6*′* are near ground potential. The higher potentials are represented in the figure inset by thicker lines on the device periphery. *V*_H_(*i*) becomes − *V*_H_(*i*) on current reversal.

Outside the device, the current direction *I*_T_ is labeled in the *opposite* direction to the flow of the electrons, following the convention that the current direction is that which a *positive* charge would take. We cannot assume positive charge carriers inside the device, since this would yield an incorrect sign for the Hall voltage.

Shaded curves within the device show the current flow pattern for this case. The current enters one corner of the device, and exits at the opposite corner. The flow direction reverses on current reversal. Current enters and exits the other two diagonal corners on magnetic field reversal. Shaded arrows pointing in the opposite direction to *I*_T_ indicate the electron motion, and are reminders to the reader that the current within the device is actually composed of electrons moving through the 2DEG in the opposite direction of the conventional current direction. The *x* axis of our coordinate system is directed along the device, with the *y* axis pointing across the device.

Fig. 1 also shows our equivalent circuit of the device. The device contact pads provide electrical access to the 2DEG at the source S*′*, the drain D*′*, and the potential pads 1*′* through 6*′*. Each contact pad is located at the end of an arm of the QHE device. Every arm in the equivalent circuit has an intrinsic resistor whose value is *R*_H_*(i)*/2. We assume that the device is *homogeneous*, i.e., that: (a) the quantized Hall resistances *R*_H_*(i)* are *all* measured on plateau regions; (b) their values are the *same* on all the Hall potential probe sets; and (c) they are all measured at the *same* magnetic flux density value *B*. The values of *R*_H_*(i) can*, however, vary with temperature [18] and current [19] as long as they are the same for each Hall probe set.

The values of *R*_H_*(i)* could also vary with frequency, but our calculations of the intrinsic impedance of the 2DEG due to the internal Hall capacitance across the QHE device [20] predicts a negligible dependence of *R*_H_*(i)* itself. We therefore greatly simplify the model, and assume temperature and current dependent *dc values* for the *R*_H_*(i)*/2 resistances in Fig. 1 and throughout this paper. (Intrinsic in-phase frequency-dependent corrections to the dc values of *R*_H_*(i)* could be inserted into our final equations if these corrections are later found to be significant, and if their algebraic relationships are known.)

The potentials at the ends of the arms at contact pads S*′*, 1*′* through 6*′*, and D*′* are produced by diamond-shaped arrays of voltage generators. Ricketts and Kemeny [21] first used these diamond arrays in equivalent circuits. Then Delahaye [22], and later Jeffery, Elmquist, and Cage [14] used ring-shaped voltage generator arrays. Although both types of arrays give essentially identical results [13], our calculations are much simpler with the diamond arrays when longitudinal resistances are included in the circuits [13]. We therefore use diamond arrays.

A voltage generator, labeled *V*_AB_ and located between a pair of arms A and B in the circuit, produces a voltage defined as [21]

$$V_{\text{AB}}\equiv \frac{R_{H}(i)}{2}|I_{A}\pm I_{B}|,$$ (1)

where *I*_A_ and *I*_B_ are the magnitudes of the currents flowing in arms A and B of the circuit. The currents *I*_A_ and *I*_B_ within the absolute quantity sign of Eq. (1) are *added* if they both enter or both leave the voltage generator, and are *subtracted* if one current enters and the other current leaves the generator. Since the voltages produced by the generators are functions of *R*_H_*(i)*, their values can vary with temperature and current. The arms A and B in the diamond-array voltage generator definitions can be the external arms S*′*, 1*′* through 6*′*, and D*′*, *or* the four internal segments containing longitudinal resistances *r*_a_, *r*_b_, *r*_c_, and *r*_d_ (which will be described later in this section).

We assume in the simple case of Fig. 1 that there are no currents in potential arms 1*′* through 6*′*, so *I*_a_ = *I*_b_ = *I*_c_ = *I*_d_ = *I*_T_. Since there are no currents in the potential arms, this means that all of the voltage generators shown in Fig. 1 have magnitudes *V*_AB_ =[ *R*_H_*(i)*/2]|*I*_T_|. For clarity, the voltage generators are indicated in the figure as batteries whose positive terminals are oriented to give the correct potentials at the end of each arm. As an example: if the potential at contact pad S*′* is 0, then the potential *V*_5′_ at contact pad 5*′* of the quantum Hall effect device is then *V*_5′_ = [*R*_H_*(i)*/2]*I*_T_ + *r*_d_*I*_T_ + [*R*_H_*(i)*/2]|*I*_T_| = [*R*_H_*(i)* + *r*_d_]*I*_T_ = *V*_H_(*i*) + *r*_d_*I*_T_; and the potential *V*_6′_ at contact pad 6*′* is *V*_6′_ = [*R*_H_*(i)*/2]*I*_T_ + *r*_d_*I*_T_ − [*R*_H_*(i)*/2]|*I*_T_| = *r*_d_*I*_T_.

The applied ac current *I*_T_ alternates direction, so the voltage generators reverse sign each half-cycle. Thus, for the part of the period in which *I*_T_ flows in the direction indicated in Fig. 1, the voltage generators have the polarities shown. Half a period later *I*_T_ changes direction, and all the voltage generators reverse polarities.

The circuit elements *r*_a_, *r*_b_, *r*_c_, and *r*_d_ in Fig. 1 represent real (in-phase) longitudinal resistances within the device. Longitudinal resistance values are obtained by potential difference measurements along a side of the device in the *x* direction. For example, the longitudinal resistance *R_x_*(2*′*,6*′*) between contact pads 2*′* and 6*′* is

$$R_{x}(2^{\prime },6^{\prime })\equiv \frac{V_{x}(2^{\prime },6^{\prime })}{I_{T}}=\frac{\left[V_{2^{\prime }}-V_{6^{\prime }}\right]}{I_{T}}.$$ (2)

where *V_x_*(2*′*,6*′*) is the voltage difference measured between contact pads 2*′* and 6*′*. Referring to Fig. 1, *V**_x_*(2*′*,6*′*) = *V*_6c_ + *r*_c_*I*_T_ − *V*_c4_ + *V*_4b_ + *r*_b_*I*_T_ − *V*_b2_. Since no current flows through arms 2*′*, 4*′*, or 6*′* in this example, *V*_6c_= *V*_c4_ = *V*_4b_ = *V*_b2_ = [*R*_H_*(i)*/2]|*I*_T_|. Therefore

$$R_{x}(2^{\prime },6^{\prime })=R_{x}(1^{\prime },5^{\prime })=r_{b}+r_{c}.$$ (3)

We will discuss the longitudinal resistances further in the next section, but note here that their values can be temperature [18] and current [19] dependent.

The quantized Hall resistance *R*_H_(3*′*,4*′*) measured between contact pads 3*′* and 4*′* in Fig. 1 is

$$R_{H}(3^{\prime },4^{\prime })\equiv \frac{V_{H}(3^{\prime },4^{\prime })}{I_{T}}=\frac{\left[V_{3^{\prime }}-V_{4^{\prime }}\right]}{I_{T}}=\frac{\left[V_{c4}+V_{c3}\right]}{I_{T}}=R_{H}(i).$$ (4)

The device shown in Fig. 1 is *homogeneous*, i.e., the quantized Hall resistances *R*_H_ are *all* measured on plateau regions, their values are the *same* between *all* the Hall potential probe sets, and they are all measured at the *same* magnetic flux density. Therefore

$$R_{H}(1^{\prime },2^{\prime })=R_{H}(3^{\prime },4^{\prime })=R_{H}(5^{\prime },6^{\prime })=R_{H}(i).$$ (5)

Note once again that *R*_H_*(i) can* be a function of temperature and current, and *can* differ from the ideal value *h*/(*e*^2^*i*).

The circuit in Fig. 1 represents a QHE device immersed in liquid helium and cooled well below 4.2 K, so that the temperature dependence corrections to *R*_H_*(i)* are negligible or small. It would be nice if this circuit was all that was required when using the device as an ac resistance standard, but life is not so simple. There are large parasitic impedances within the ac standard that can significantly affect the measured values of the ac quantized Hall resistance. The next section describes the ac quantized Hall resistance standard and the parasitic impedances.

## 5. Equivalent Electrical Circuit of an AC QHE Standard

The quantized Hall resistance *R*_H_*(i)* of an ac QHE resistance standard (ac QHRS) can be experimentally compared with the impedances of ac reference standards using ac measurement systems. Like other laboratories, NIST plans to use ac resistors as reference standards, and an ac ratio bridge measurement system to make the comparisons.

**Fig. 2:**
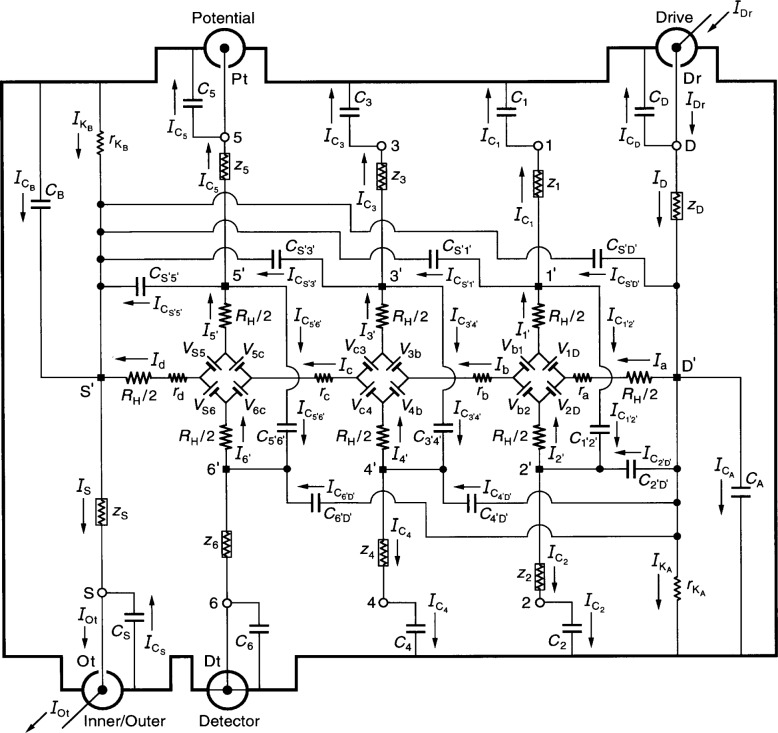
An equivalent electrical circuit representation of an ac QHE resistance standard with single-series “offset” connections to the device. The symbols are explained in Sec. 5. See Secs. 6.1 to 6.3 for the circuit analysis. The quantized Hall resistance is being measured in an ac ratio bridge using four-terminal-pair [23,24] measurement techniques. The ac ratio bridge is not shown in the figure, nor is the ac reference impedance standard with which the QHE standard is being compared.

Figure 2 shows an equivalent electrical circuit representation of an ac QHRS in which the QHRS is being measured with an ac bridge using four-terminal-pair [23,24] techniques. We use four-terminal-pair techniques so that error contributions from the ac QHRS, the ac reference standard, and the ac bridge can all be analyzed separately. *Neither* the ac reference standard *nor* the ac ratio bridge are shown in the figure. This circuit of an ac QHRS is rather detailed, so we explain it one step at a time.

The ac QHRS of Fig. 2 is bounded by an electrical shield indicated schematically by thick lines. Actual shields have complicated surface geometries. They consist of: (a) conductive surfaces surrounding the QHE device and its sample holder at liquid helium temperatures; (b) the outer conductors of eight coaxial leads within the sample probe; and (c) the outer conductors of eight coaxial leads extending from the top of the sample probe to room temperature access points S, 1 through 6, and D. (The electrical shields will also be referred to in the text as “outer conductors”.) To simplify the figure, we label only currents in the inner conductors.

The ac QHRS has electrical access at room temperature via four coaxial measurement ports labeled Inner/Outer, Detector, Potential, and Drive. These four ports are used in the four-terminal-pair measurements [23,24], where each coaxial port is referred to as a “terminal-pair”. The four coaxial ports are connected to room temperature access points S, 6, 5, and D in the figure. (We will explain in Sec. 6 why access points 6 and 5 were chosen rather than points 4 and 3.)

Proper four-terminal-pair measurements are not easy, and we will discuss only the essential requirements. A great advantage of using this measurement technique is that the ac QHRS can be analyzed *separately* from the ac ratio bridge and from the ac reference standard.

The ideal four-terminal-pair measurement definition [23,24] of *R*_H_*(i)* is satisfied by the following three simultaneous conditions: (1) the current *I*_Dr_at the Drive coaxial port is adjusted so that there are *no* currents in the inner or outer conductors of the Potential coaxial port, i.e., *I*_Pt_ = 0; (2) the potential difference is *zero* across the inner and outer conductors of the Detector coaxial port; and (3) there are *no* currents in the inner or outer conductors of the Detector coaxial port, i.e., *I*_Dt_ = 0.

It is implicit in the four-terminal-pair definition that *each* coaxial port is treated as a terminal-pair, and that the current in the inner conductor of *every* port is *equal* and *opposite* to the current in the outer conductor (the shield) of that port. Coaxial chokes [25] (located outside the ac QHRS and therefore not shown in the figure) assure that this equal and opposite current condition is satisfied for each of the four terminal-pair ports in the circuit. The current *I*_Ot_exits the ac QHRS at the Inner/Outer port and enters the ac reference standard (not shown).

A “virtual” short has been drawn in Fig. 2 as a line between the shield and inner conductor at the Detector coaxial port to indicate four-terminal-pair condition number (2). We let the Detector potential be zero, i.e., *V*_Dt_ = 0.

The reader will recognize the equivalent circuit representation of the QHE device located in the central region of Fig. 2, with the same magnetic field and current directions as Fig. 1. There are now currents in the side arms of the device, so the voltages generated are different than those in Fig. 1 and Sec. 4. For example *V*_1D_ = [*R*_H_*(i)*/2]|*I*_a_ − *I*_1′_|.

The device and its contact pads S*′*, 1*′* through 6*′*, and D*′* are cooled well below 4.2 K to minimize temperature dependent effects [18]. The device is mounted in a sample holder at the bottom of the sample probe. Thin wires connect the device contact pads S*′*, 1*′* through 6*′*, and D*′* to corresponding points on the sample holder. The sample holder is not explicitly shown, so points S*′*, 1*′* through 6*′*, and D*′* in the figure can also be considered as part of the sample holder.

A coaxial lead extends from each liquid helium temperature point S*′*, 1*′* through 6*′*, and D*′* to corresponding room temperature access points S, 1 through 6, and D located outside the sample probe (but still within the ac QHRS). The inner conductor of each coaxial cable has a resistance *r*_S_, *r*_1_ through *r*_6_, or *r*_D_. This resistance includes the contact resistance to the 2DEG, the wire resistance connecting a contact pad on the device to a point on the sample holder and then to a coaxial lead, and the inner conductor resistance of that coaxial lead. (We combine these resistances into a single resistance to make the circuit as simple as possible.)

The inner conductor coaxial lead resistances vary with the liquid helium level in the sample probe. They can be measured pair-wise (using room temperature access points S, 1 through 6, and D) as a function of liquid helium level via two-terminal dc resistance measurements by temporarily replacing the QHE device with electrical shorts at positions S*′*, 1*′* through 6*′*, and D*′*. The cooled inner conductor coaxial lead resistances are typically each about 1 Ω in ac quantized Hall resistance standards. The outer conductor coaxial lead resistances depend on the type of coaxial cable, and their values also vary with liquid helium level. The shields will be shorted together at the sample holder end of the NIST sample probes, but electrically isolated elsewhere, so their two-terminal resistances can be measured pairwise. Typical outer conductor values range between about 0.1 Ω and 1 Ω in ac quantized Hall resistance sample probes.

Each sample probe lead has an inner conductor inductance *L*_S_, *L*_1_ through *L*_6_, or *L*_D_ that is electrically connected in series with the lead resistances *r*_S_, *r*_1_ through *r*_6_, or *r*_D_, producing lead impedances *z*_S_, *z*_1_ through *z*_6_, or *z*_D_, where

$$z_{S}=r_{S}+j\omega L_{S},$$ (6)

etc. (We use the symbol j, rather than the symbol i, to denote the 90° out-of-phase component of *z*_S_ in Eq. (6) to avoid confusion with the quantum Hall plateau number *i*.) Due to severe space limitations in Fig. 2, these impedances are unconventionally drawn as resistors within rectangles.

Just as for the lead resistances, the inductances can be measured pair-wise at room temperature access points S, 1 through 6, and D as a function of liquid helium level via two-terminal ac measurements by temporarily replacing the QHE device with electrical shorts at positions S*′*, 1*′* through 6*′*, and D*′*. The inductance of the inner conductor lead of a typical ac QHE sample probe is about 1 × 10^−6^ H. We assume that the bonding pad wires at the device are thick enough to not significantly vibrate in the magnetic field when ac currents flow through them [4], but (if necessary) the out-of-phase “inductance” generated by this resonant frequency vibration [4] could be included in the lead inductances.

The eight coaxial leads, labeled S, 1 through 6, and D, each have an inner and an outer conductor. The outer conductors of the coaxial leads will be connected together outside the sample probe to help satisfy the four-terminal-pair measurement conditions. As mentioned earlier, the outer conductors of these leads act as electrical shields, and are represented schematically as thick lines in Fig. 2. Other outer conductors of the ac QHRS also contribute to the thick lines: for example, the shields around the QHE device and the sample holder.

Large capacitances-to-shield, labeled as *C*_S_, *C*_1_ through *C*_6_, and *C*_D_, exist between the inner and outer conductors of these eight coaxial leads. This large capacitance is unavoidable, and is determined mainly by the sample probe length. However, the coaxial cable length outside the sample probe to the four coaxial four-terminal-pair measurement ports labeled Inner/Outer, Detector, Potential, and Drive can be (and should be) kept as short as possible.

The open-circuit capacitances can be individually measured at room temperature access points S, 1 through 6, and D as a function of liquid helium level by temporarily removing the QHE device from the sample probe at the points S*′*, 1*′* through 6*′*, and D*′*. The capacitance-to-shield of each coaxial lead is about 250 pF in typical ac QHE sample probes, but it should be reduced to nearer 100 pF (1 × 10^−+″^ F) in a short sample probe being designed at NIST.

A predominately 90° out-of-phase current $I_{C_{S}}$, $I_{C_{1}}$through $I_{C_{6}}$, or $I_{C_{D}}$flows through each coaxial lead. These currents, and all the other currents in Fig. 2, have the correct signs for their dominant phase components in the half-cycle of the Drive current under consideration. The signs are verified in Sec. 6, where it is found that the major components of all currents shown in the figure have positive signs for this half-cycle.

The coaxial leads are not the only sources of capacitances-to-shield. There are also additional contributions between the QHE device—sample holder combination and the electrical shielding surrounding them. These additional capacitances-to-shield are labeled *C*_A_ and *C*_B_ in Fig. 2, where they are placed at either end of the QHE device. We will make these two capacitances as small as possible in the NIST sample probes by: (a) mounting the QHE devices on electrically insulated surfaces; (b) using electrically insulated sample holders; and (c) completely surrounding the QHE device—sample holder combination with a cylindrically-shaped “pillbox” shield located as far as possible from the QHE device and its holder.

The additional capacitances *C*_A_ and *C*_B_ can be determined by two methods. In the first method the magnetic field is adjusted so the QHE device is on a QHE plateau. The external Drive and Inner/Outer coaxial leads are removed from the ac QHRS, and an applied voltage signal is placed across the inner and outer conductors of the Drive port. A measured voltage signal appears across the inner and outer conductors of coaxial leads S, D, 1, 3, and 5 for the magnetic field direction assumed in Fig. 2, so these particular coaxial leads draw most of the 90° out-of-phase current. Therefore the measured total capacitance-to-shield *C*_T_ is approximately *C*_T_(*B*) ≈ *C*_1_ + *C*_3_ + *C*_5_ + *C*_D_ + *C*_A_, and the value of *C*_A_ can be obtained by subtracting the value of *C*_1_ + *C*_3_ + *C*_5_ + *C*_D_from *C*_T_ (*B*). The magnetic field is reversed. Then *C*_T_(−*B*) ≈ *C*_2_ + *C*_4_ + *C*_6_ + *C*_D_ + *C*_B_ when the applied voltage signal is placed across the inner and outer conductors at the Inner/Outer port, thus yielding the value of *C*_B_. We expect both *C*_A_ and *C*_B_will be about 1 pF or smaller in the NIST sample probes, so both *C*_T_ (*B*) and *C*_T_(−*B*) need to be measured to within at least 1 pF.

In the second method of measuring *C*_A_ and *C*_B_, the magnetic flux density *B* is reduced to zero. The quantum Hall voltages disappear, so the voltage generators in Fig. 2 can be replaced in the equivalent circuit by electrical shorts. The QHE device can now be modeled as a two-dimensional sheet resistance, and the *R*_H_*(i)*/2 resistances located at the source and drain ends of the QHE device are zero. Longitudinal resistances *r*_a_, *r*_b_, *r*_c_, and *r*_d_ become much larger than they were when on a QHE plateau. Their values can be obtained by four-terminal dc resistance measurements in a sample probe having a pair of leads to the source and another pair of leads to the drain. The *R*_H_*(i)*/2 resistances of the six QHE side arms in Fig. 2 are replaced by much smaller resistances whose values can be obtained from two-terminal measurements via room temperature access points S, 1 through 6, and D once the appropriate lead and longitudinal resistances are subtracted. An applied voltage signal placed across the inner and outer conductors of the Drive port would cause a voltage signal to appear across the inner and outer conductors of all the capacitances-to-shield. Thus the total capacitance-to-shield is given by the algebraic expression *C*_T_ = *C*_S_ + *C*_1_ + *C*_2_ + *C*_3_ + *C*_4_ + *C*_5_ + *C*_6_ + *C*_D_ + *C*_A_ + *C*_B_, where *C*_A_ ≈ *C*_B_ if the QHE device, the sample holder, and the bonding wires between them are all symmetrically arranged. We again expect both *C*_A_ and *C*_B_ will be about 1 pF or smaller in the NIST sample probes.

The equivalent circuit of Fig. 2 also accounts for leakage currents between the ac QHRS’s inner conductors and the shields via resistances $r_{K_{A}}$ and $r_{K_{B}}$ located on either side of the QHE device. Rather large voltages are used when measuring leakage resistances, so it would be safest to temporarily replace the QHE device with shorts when measuring the total open-circuit leakage resistance *r*_Lk_ at access point S, 1 through 6, or D. If the leakage resistances are symmetrically distributed, then $r_{K_{A}}\approx r_{K_{B}}\approx 2r_{\text{Lk}}$. (Their values are large compared with the lead resistances, so they are essentially connected in parallel within the circuit.) The NIST sample probes will be constructed so these leakage resistances are very large; $r_{K_{A}}$ and $r_{K_{B}}$ should be at least 10^4^ Ω, but in the numerical examples of this paper we will assume a value of 10^12^ Ω due to dirty coaxial connectors.

At bridge balance the ac quantized Hall voltage *V*_H_(*i*) = *V*_H_(5,6) = *V*_Pt_ is defined as

$$V_{\text{Pt}}=V_{H}(5,6)=[1+\Delta_{56}]R_{H}(i)I_{\text{Ot}},$$ (7)

where Δ_56_ is the correction factor to *R*_H_*(i)* to be determined in this analysis. Please recall from Sec. 4 that we assume that *R*_H_*(i)* can be a function of temperature and current, and can therefore differ from the ideal value *h*/(*e*^2^*i*). Measured values of *V*_Pt_ vary with frequency [4–9], but it is not clear whether this is due to a frequency dependence of *R*_H_*(i)*, of Δ_56_, or of both *R*_H_*(i)* and Δ_56_. Since our calculations of the intrinsic impedance of the 2DEG due to the internal Hall capacitance across the QHE device [20] predict a negligible internal capacitance of the 2DEG itself, we assume that *R*_H_*(i)* is not frequency dependent, and that the dc values are appropriate for the *R*_H_*(i)*/2 resistances in the figure. (This assumption does not preclude adding a frequency dependence to the measured dc values of *R*_H_*(i)* if it is found to be necessary.)

The symbols *r*_a_, *r*_b_, *r*_c_, and *r*_d_ in Fig. 2 represent real (in-phase) longitudinal resistances within the QHE device. Their measured dc values can vary with temperature [18] and current [19]. Sample probes normally used in dc QHE measurements have ten leads, with a pair of leads to the source contact pad S*′* and another pair to the drain contact pad D*′*. Only one lead of each pair carries the current, so all four dc longitudinal resistances *r*_a_, *r*_b_, *r*_c_, and *r*_d_ can be obtained using four-terminal dc measurements.

In order to reduce the heat load on the liquid helium, sample probes for the ac QHE usually have a single coaxial lead to each of the eight contact pads. Therefore only *r*_b_ and *r*_c_ can be determined directly via four-terminal-pair ac measurements. For example, a four-terminal-pair ac longitudinal resistance measurement of *r*_c_ could be made by moving the Potential coaxial port from room temperature access point 5 to point 4 in Fig. 2, and measuring the ac longitudinal voltage *V_x_*(4,6)

$$V_{x}(4,6)=[1+\Delta_{46}]r_{c}I_{\text{Ot}},$$ (8)

where Δ_46_ is the correction factor to *r*_c_to be determined in this analysis. Values for *r*_a_and *r*_c_could be estimated from their dc *r*_a_/*r*_b_and *r*_d_/*r*_c_ratios if the measured *r*_b_/*r*_c_ratio happens to be the same for both ac and dc measurements using the same sample probe during the same cool-down. We are now considering using a pair of coaxial leads to the source contact pad S*′* and another pair of coaxial leads to the drain contact pad D*′* in at least one NIST ac sample probe. That would allow ac and dc measurements of all *r*_a_, *r*_b_, *r*_c_, and *r*_d_values on the same cool-down.

Our analyses do not include this possibility of a pair of coaxial leads to the source and another pair to the drain. It would take much work to explicitly include them in new calculations; however, we can account for the presence of these two additional coaxial leads in the analysis by including lead resistances *r*_A_and *r*_B_, and lead inductances *L*_A_and *L*_B_in current paths *C*_A_and *C*_B_of Fig. 2, and adding two room temperature access points A and B. (The paths would be analogous to those through capacitors *C*_S_, *C*_1_, *C*_2_, *C*_3_, *C*_4_, *C*_5_, *C*_6_, and *C*_D_). By measuring the lead resistances *r*_A_and *r*_B_, and the lead inductances *L*_A_and *L*_B_, the values of *C*_A_and *C*_B_in Fig. 2 could be replaced with

$${\tilde{C}}_{A}=\frac{C_{A}}{\left[(1-\omega^{2}C_{A}L_{A})+j\omega C_{A}r_{A}\right]}$$ (9a)

and

$${\tilde{C}}_{B}=\frac{C_{B}}{\left[(1-\omega^{2}C_{B}L_{B})+j\omega C_{B}r_{B}\right]}.$$ (9b)

The capacitance-to-shield contributions between the QHE device—sample holder combination and the electrical shielding surrounding them would no longer be accounted for, so one-tenth of these additional capacitances would need to be added to each of the ten coaxial lead capacitances *C*_S_, *C*_B_, *C*_1_ through *C*_6_, *C*_A_, and *C*_D_.

With one exception [26], the reported ac longitudinal resistances obtained from the real, in-phase components of the ac longitudinal voltage measurements are significantly larger than the dc longitudinal resistances in the same device under the same temperature and magnetic field conditions. The measured ac longitudinal resistances increase with increasing frequency of the applied current, and are of order 1 mΩ at 1592 Hz [4,5,11]. The large ac longitudinal voltages might be due to intrinsic frequency dependences of *r*_a_, *r*_b_, *r*_c_, and *r*_d_within the device, to corrections such as Δ_46_ of Eq. (8) via parasitic impedances of the QHRS, or to both of them. Calculations of the kinetic inductance of the 2DEG [20] and the magnetic inductance of the device [20] provide no plausible explanations via intrinsic impedance for significant frequency dependences of *r*_a_, *r*_b_, *r*_c_, and *r*_d_, suggesting that the frequency dependence of the ac longitudinal resistance is due to parasitic impedances of the ac QHRS, and therefore arises from correction factors such as Δ_46_. We will use numerical calculation examples in this paper that assume either: (a) dc values for *r*_a_, *r*_b_, *r*_c_, and *r*_d_; or (b) the worst-case scenario in which they are frequency dependent and have 1 mΩ values at 1592 Hz.

All the circuit components described so far were included in the analyses of Ref. [16]. We now discuss the wire-to-wire capacitances shown in Fig. 2 and labeled as *C*_1′2′_, *C*_2′D′_, *C*_S′D′_, etc. Primes are used in these labels because the capacitors are located in the QHE device and sample holder region of the circuit near S*′*, 1*′* through 6*′*, and D*′*. We place them there because the outer conductors of the coaxial shields should completely surround the inner conductors elsewhere in the circuit, and thus yield negligible contributions to the wire-to-wire capacitances.

The circuit is complicated enough, so only those wire-to-wire capacitors that have quantum Hall voltages on one side of the capacitors and ground potentials on the other side are shown in the figure because those capacitors draw the largest out-of-phase currents. We therefore exclude capacitances such as *C*_5′3′_, *C*_3′1′_, and *C*_1′D′_ that essentially have quantum Hall voltages on both sides of the capacitors, and capacitances such as *C*_4′2′_, *C*_6′4′_, and *C*_S′6′_ that essentially have ground potentials on both sides.

Each wire-to-wire capacitance has contributions from: (a) metallic contact pads on the QHE device; (b) wires connecting those contact pads to points on the sample holder (and to any intermediate bonding pads); and (c) metallic surfaces within the sample holder if those surfaces are not surrounded by coaxial shields. The wire-to-wire capacitance values depend on distances between the pairs of wires, conducting surfaces, or bonding pads, so we will try to physically separate them as much as possible. These capacitances will each have different values in the algebraic solutions since *C*_S′5′_ > *C*_S′3′_ > *C*_S′1′_ > *C*_S′D′_ and *C*_2′D′_ > *C*_4′D′_ > *C*_6′D′_ because of increasing physical separations, but (for simplicity) we will assign equal values to them in the numerical examples. These equal values will usually be assigned 0.1 pF in the calculations, exceeding the maximum values expected in NIST sample probes.

Some laboratories have tried reducing the wire-to-wire capacitances by using evaporated metallic guard electrodes on the device mounting surfaces to isolate the quantum Hall voltages from the ground potentials. These guard electrodes have very little effect since the dielectric constant is about 13 times greater in GaAs than in liquid helium; the electric field lines are thus concentrated within the device and are not significantly blocked by the surface guard electrodes.

The individual wire-to-wire capacitance values can be obtained by (1) adjusting the magnetic flux density *B* so that the allowed states of the Landau levels are completely filled and the QHE device is operating on a plateau; (2) removing the applied Drive current; and (3) making two-terminal ac measurements at room temperature access ports S, 1 through 6, and D. The Hall capacitances across the device *C*_1′2′_, *C*_3′4′_, and *C*_5′6′_ have the contributions (a), (b), and (c) mentioned two paragraphs above, but they also have contributions from the intrinsic capacitance of the 2DEG. We predicted that this internal Hall capacitance was negligible [20], but its actual contribution can be measured by observing any increase in *C*_1′2′_, *C*_3′4′_, or *C*_5′6′_ values when the Drive current *I*_Dr_ is turned on.

The capacitances, inductances, lead resistances, contact resistances, and leakage resistances of Fig. 2 contribute parasitic impedances to measurements of the ac QHRS. They are drawn as discrete circuit elements. In reality they are distributed within the standard, and could, in principle, be better represented. For example, we could replace capacitance-to-shield *C*_1_ with a capacitor of value *C*_1_/2, then place a second capacitor of value *C*_1_/2 and a series-connected outer shield impedance $z_{1}^{\prime }$ between the other side of circuit element *z*_1_ at point 1*′* and the first *C*_1_/2 capacitor. This distributed series-parallel impedance would, however, greatly complicate the circuit analyses, with an insignificant gain in accuracy. (Our discrete-elements circuit slightly over-emphasizes the capacitance-to-shield currents if $z_{1}\geq z_{1}^{\prime }$ and gives the same capacitance-to-shield currents if $z_{1}=z_{1}^{\prime }$.)

This completes the description of the equivalent circuit. The next section analyzes the circuit.

## 6. Analysis of the Single-Series “Offset” Circuit

We refer to the circuit in Fig. 2 as single-series “offset”: *single-series* because there is just *one* current lead connected to the source contact pad S*′*, and just *one* current lead connected to the drain pad D*′* of the QHE device; and “*offset*” because the Hall voltage leads are connected to arms 5 and 6 of the device, rather than to the central arms 3 and 4. Arms 5 and 6 are those closest to the source contact pad S*′*, which is at a potential near that of the circuit ground. Arms 5 and 6 are also nearest to the ac reference resistor (not shown in the figure).

This single-series “*offset*” circuit is considered here because only it, and the quadruple-series circuit investigated in Sec. 7, provided the desired accuracy in earlier stages of the analysis [13,16] with all sample probe leads attached. Other circuit connection possibilities had been eliminated in Refs. [13] and [16] due to undesirable parasitic impedance effects.

### 6.1 Exact Single-Series “Offset” Equations

We use Kirchoff’s rules to sum the voltages around loops and the currents at branch points to obtain exact algebraic equations for the equivalent electrical circuit shown in Fig. 2. Four of the current solutions are trivial because of the four-terminal-pair definition [23,24] listed in Sec. 5

$$I_{\text{Dt}}=I_{\text{Pt}}=I_{C_{6}}=I_{z_{6}}=0.$$ (10)

Those four currents are not included in the 35 inner conductor currents shown in the figure since they are assumed to be zero in the ideal four-terminal-pair definition. (They can be adjusted to be zero within several parts in 10^9^ of *I*_Ot_ in NIST bridges.) The remaining circuit has 21 independent voltage loop equations and 13 independent current branch points. This gives a set of 34 coupled equations for the 35 non-zero currents. Our goal is to simultaneously solve this set of 34 coupled equations, and to then express all the currents, and the quantum Hall voltage, in terms of *I*_Ot_ because that is the current which enters the ac reference standard (not shown in Fig. 2).

Finding the exact algebraic equations for all the currents, and for the correction factor Δ_56_ to the quantum Hall voltage as defined by Eq. (7), is difficult because of the many coupled equations. However, it is important to obtain the *exact* solutions, rather than initially guess approximate solutions, because the frequency-dependent effects we are trying to minimize or eliminate are small, but significant.

The algebraic derivations were done “by hand”, rather than with computer software programs. Doing it “by hand” allowed us to investigate each stage of the solution, and to get a physical sense of the equations. Computer software programs generated pages of equations, with no simplification and no physical insight. Computer programs were, however, used to calculate numerical examples.

The following procedures were used to find the exact solutions: (a) two of the authors independently derived the equations. They made the same choices of current branch points, but different choices of paths for the independent voltage loops and the substitutions of algebraic variables. A current branch point example is

$$I_{C_{S}}=I_{\text{Ot}}-I_{S}.$$ (11)

A voltage loop example around the path *C*_S_, *S′*, *C*_B_, and back through the shield to *C*_S_is

$$-\frac{1}{j\omega C_{S}}I_{C_{S}}+z_{S}I_{S}+\frac{1}{j\omega C_{B}}I_{C_{B}}=0$$ (12a)

$$I_{C_{B}}=\frac{C_{B}}{C_{S}}I_{C_{S}}-j\omega C_{B}z_{S}I_{S}$$ (12b)

$$I_{C_{B}}=A_{1}I_{C_{S}}-A_{2}I_{S}.$$ (12c)

Liberal use was made of algebraic substitutions such as *A*_1_ and *A*_2_ of Eq. (12c). This added to the “bookkeeping” problems, but reduced the possibilities for algebraic errors as the equations were uncoupled.

The 34 branch and loop equations contained 35 coupled currents. The two authors used different strategies to “decouple” these 34 initial equations and thereby derive the results; (b) they then independently used Mathcad PLUS 6 (Professional Edition for Macintosh computers)^1^ software to enter their own algebraic substitutions and their own current equations, and then compared numerical results with the other author. The numerical results for the currents were in exact agreement to within at least six significant figures when using the highest allowed precision of the Mathcad software when the values were expressed in the scientific notation 1.23456 × 10^−yz^; (c) the third author took the two initial sets of 34 coupled current equations derived by the other two authors, entered the equations in matrix form into the Mathcad software, and for particular numerical examples, inverted the matrices to solve for the currents. This method for solving the coupled equations is faster, but less reliable, than solving the equations “by hand”, since round-off errors can go undetected. However, most results agreed with the numerical results of the other two authors to within four to six significant figures. The largest difference from the exact solutions discussed below was only a 1 part in 10^11^ discrepancy in the out-of-phase (imaginary) term of $I_{C_{B}}$; and finally (d) the exact algebraic solutions discussed below were then checked at least twice by all three authors, so we are reasonably confident of the equations.

In the exact solutions discussed here, all 35 coupled currents in the 34 initial equations were expressed in terms of the “core” currents *I*_Ot_, $I_{C_{S}}$, *I*_S_, $I_{C_{B}}$, *I*_d_, *I*_c_, *I*_b_, *I*_a_, $I_{C_{A}}$, and *I*_D_. These “core” currents were then successively substituted into the remaining equations to obtain the equation

$$I_{S}=k_{8}I_{\text{Ot}},$$ (13)

which looks “simple”, except that the substitution variable *k*_8_ is a function of 203 other algebraic substitutions. The remaining currents were easily obtained once Eq. (13) was solved. As mentioned above, the numerical results agree with the results of the other exact solution to within six significant figures, and also agree very well with the matrix inversion.

In addition, the exact solution results agree with the numerical results listed in Eqs. (20) and (21) of Ref. [16] to within at least six significant figures. The calculations of Ref. [16] did not include wire-to-wire capacitances. (Some of the algebraic substitutions of the present work approach infinity as the wire-to-wire capacitances approach zero, so a limiting value of 10^−60^ F was assigned to each wire-to-wire capacitance to avoid this problem. The 10^−60^ F value was chosen because there were no differences in the numerical results when the wire-to-wire capacitances were within the range 10^−40^ F to 10^−80^ F.)

The exact equations for the currents and the quantum Hall voltage were published in our earlier analyses [13,16]. That is not feasible here because: (a) the chances for error are too numerous; and (b) there are so many algebraic substitutions that the final answers convey no physical insight. Instead, we: (1) present numerical examples of the currents and quantum Hall voltage in Sec. 6.2; (2) list the approximate equations for the currents and quantum Hall voltage in Sec. 6.3; and (3) can supply the reader with a 30 page computer printout which lists the 204 algebraic substitutions, the exact current and quantum Hall voltage equations, the approximate equations, and numerical calculations for a particular set circuit component values of the reader’s choice.

### 6.2 Numerical Examples

We give here numerical examples of how the parasitic impedances within the ac QHRS affect the currents and the measured values of *V*_H_(5,6). Cardinal numbers are usually assigned to the circuit element components of Fig. 2 in the examples to emphasize that the results presented below are not intended as calculations for a particular sample probe. We will measure the actual values of all the parasitic impedance components of our sample probes as a function of liquid helium level.

Both the *i* = 2 (12 906.4 Ω) and *i* = 4 (6 453.2 Ω) plateaus can be measured in ac experiments, so let *R*_H_ = 10 000 Ω. The cardinal values we usually use in the examples are

$$R_{H}=10^{4}\Omega$$ (14a)

$$r_{S}=r_{1}=r_{2}=r_{3}=r_{4}=r_{5}=r_{6}=r_{D}=1\;\Omega$$ (14b)

$$r_{a}=r_{b}=r_{c}=r_{d}=10^{-3}\;\Omega$$ (14c)

$$r_{K_{A}}=r_{K_{B}}=10^{12}\;\Omega$$ (14d)

$$C_{S}=C_{1}=C_{2}=C_{3}=C_{4}=C_{5}=C_{6}=C_{D}=10^{-10}F$$ (14e)

$$C_{A}=C_{B}=10^{-12}F$$ (14f)

$$C_{1^{\prime }2^{\prime }}=C_{3^{\prime }4^{\prime }}=C_{5^{\prime }6^{\prime }}=C_{S^{\prime }D^{\prime }}=C_{S^{\prime }1^{\prime }}=C_{S^{\prime }3^{\prime }}=C_{S^{\prime }5^{\prime }}=C_{2^{\prime }D^{\prime }}=C_{4^{\prime }D^{\prime }}=C_{6^{\prime }D^{\prime }}=10^{-13}F$$ (14g)

$$L_{S}=L_{1}=L_{2}=L_{3}=L_{4}=L_{5}=L_{6}=L_{D}=10^{-6}H$$ (14h)

$$\omega =10^{4}\text{rad}/s.$$ (14i)

Note that the 100 pF capacitances-to-shield values of Eq. (14e) may be close to those that will be obtained in the short NIST sample probe, but typical ac probes have values around 250 pF. Note also that *C*_S′5′_ > *C*_S′3′_ > *C*_S′1′_ > *C*_S′D′_ and *C*_2′D′_ > *C*_4′D′_ > *C*_6′D′_ in real sample probes because of increasing physical separations between corresponding pairs of inner conducting surfaces.

Using these cardinal numbers, and grouping similar types of currents, the numerical results for the currents shown in Fig. 2 are

$$I_{S}=\{[1.00000]-j[1.0\times 10^{-6}]\}I_{\text{Ot}}$$ (15a)

$$I_{d}=\{[1.00000]-j[4.1\times 10^{-5}]\}I_{\text{Ot}}$$ (15b)

$$I_{c}=\{[1.00000]+j[0.00996]\}I_{\text{Ot}}$$ (15c)

$$I_{b}=\{[0.99990]+j[0.01996]\}I_{\text{Ot}}$$ (15d)

$$I_{a}=\{[0.99970]+j[0.02996]\}I_{\text{Ot}}$$ (15e)

$$I_{D}=\{[0.99970]+j[0.03010]\}I_{\text{Ot}}$$ (15f)

$$I_{\text{Dr}}=\{[0.99940]+j[0.04010]\}I_{\text{Ot}}$$ (15g)

$$I_{K_{B}}=\{-[2.0\times 10^{-15}]+j[2.0\times 10^{-13}]\}I_{\text{Ot}}$$ (15h)

$$I_{K_{A}}=\{[1.0\times 10^{-8}]+j[3.0\times 10^{-10}]\}I_{\text{Ot}}$$ (15i)

$$I_{C_{B}}=\{-[2.0\times 10^{-9}]-j[2.0\times 10^{-11}]\}I_{\text{Ot}}$$ (15j)

$$I_{C_{A}}=\{-[3.0\times 10^{-6}]+j[1.0\times 10^{-4}]\}I_{\text{Ot}}$$ (15k)

$$I_{C_{S}}=\{-[2.1\times 10^{-7}]+j[1.0\times 10^{-6}]\}I_{\text{Ot}}$$ (15l)

$$I_{C_{4}}=\{-[2.0\times 10^{-7}]-j[1.0\times 10^{-9}]\}I_{\text{Ot}}$$ (15m)

$$I_{C_{2}}=\{-[4.0\times 10^{-7}]-j[1.0\times 10^{-9}]\}I_{\text{Ot}}$$ (15n)

$$I_{C_{5}}=\{[6.2\times 10^{-7}]+j[0.01000]\}I_{\text{Ot}}$$ (15o)

$$I_{C_{3}}=\{-[1.0\times 10^{-4}]+j[0.01000]\}I_{\text{Ot}}$$ (15p)

$$I_{C_{1}}=\{-[2.0\times 10^{-4}]+j[0.01000]\}I_{\text{Ot}}$$ (15q)

$$I_{C_{D}}=\{-[3.0\times 10^{-4}]+j[0.01000]\}I_{\text{Ot}}$$ (15r)

$$I_{6^{\prime }}=\{-[3.0\times 10^{-7}]+j[2.0\times 10^{-5}]\}I_{\text{Ot}}$$ (15s)

$$I_{4^{\prime }}=\{-[2.0\times 10^{-7}]+j[2.0\times 10^{-5}]\}I_{\text{Ot}}$$ (15t)

$$I_{2^{\prime }}=\{-[1.0\times 10^{-7}]+j[2.0\times 10^{-5}]\}I_{\text{Ot}}$$ (15u)

$$I_{5^{\prime }}=\{[6.2\times 10^{-7}]+j[0.01002]\}I_{\text{Ot}}$$ (15v)

$$I_{3^{\prime }}=\{-[1.0\times 10^{-4}]+j[0.01002]\}I_{\text{Ot}}$$ (15w)

$$I_{1^{\prime }}=\{-[2.0\times 10^{-4}]+j[0.01002]\}I_{\text{Ot}}$$ (15x)

$$I_{C_{6^{\prime }D^{\prime }}}=\{-[3.0\times 10^{-7}]+j[1.0\times 10^{-5}]\}I_{\text{Ot}}$$ (15y)

$$I_{C_{4^{\prime }D^{\prime }}}=\{-[3.0\times 10^{-7}]+j[1.0\times 10^{-5}]\}I_{\text{Ot}}$$ (15z)

$$I_{C_{2^{\prime }D^{\prime }}}=\{-[3.0\times 10^{-7}]+j[1.0\times 10^{-5}]\}I_{\text{Ot}}$$ (16a)

$$I_{C_{S^{\prime }5^{\prime }}}=\{[4.1\times 10^{-10}]+j[1.0\times 10^{-5}]\}I_{\text{Ot}}$$ (16b)

$$I_{C_{S^{\prime }3^{\prime }}}=\{-[1.0\times 10^{-7}]+j[1.0\times 10^{-5}]\}I_{\text{Ot}}$$ (16c)

$$I_{C_{S^{\prime }1^{\prime }}}=\{-[2.0\times 10^{-7}]+j[1.0\times 10^{-5}]\}I_{\text{Ot}}$$ (16d)

$$I_{C_{S^{\prime }D^{\prime }}}=\{-[3.0\times 10^{-7}]+j[1.0\times 10^{-5}]\}I_{\text{Ot}}$$ (16e)

$$I_{C_{5^{\prime }6^{\prime }}}=\{[6.1\times 10^{-10}]+j[1.0\times 10^{-5}]\}I_{\text{Ot}}$$ (16f)

$$I_{C_{3^{\prime }4^{\prime }}}=\{-[1.0\times 10^{-7}]+j[1.0\times 10^{-5}]\}I_{\text{Ot}}$$ (16g)

$$I_{C_{1^{\prime }2^{\prime }}}=\{-[2.0\times 10^{-7}]+j[1.0\times 10^{-5}]\}I_{\text{Ot}}.$$ (16h)

The 90° out-of-phase (imaginary or j) components of shunt currents $I_{C_{5}}$, $I_{C_{3}}$, $I_{C_{1}}$, $I_{C_{D}}$ and $I_{C_{A}}$ are much larger than for shunt currents $I_{C_{2}}$, $I_{C_{4}}$, $I_{C_{S}}$, and $I_{C_{B}}$ because contact pads 5*′*, 3*′*, 1*′*, and D*′* are all near the quantum Hall potential, rather than near the shield potential. A 1 % out-of-phase current passes through each of the coaxial cable capacitances *C*_5_, *C*_3_, *C*_1_, and *C*_D_in this example, which is not necessarily a problem if there is enough adjustment in the bridge Drive to provide this extra 4 % of out-of-phase current to *I*_Dr_.

The exact equation for the quantum Hall voltage is obtained by summing the voltages between the inner conductors of the Detector coaxial port and the Potential coaxial port. Taking the path through arm 6, voltage generators *V*_S6_ and *V*_S5_, and arm 5 we find that

$$V_{H}(5,6)=R_{H}I_{d}-R_{H}I_{6^{\prime }}-z_{5}I_{C_{5}},$$ (17a)

which can also be expressed in the form

$$V_{H}(5,6)=[1+\Delta_{56}]R_{H}I_{\text{Ot}}.$$ (17b)

The numerical results are

$$V_{H}(5,6)=\{1+[1.1\times 10^{-6}]-j[6.2\times 10^{-5}]\}R_{H}I_{\text{Ot}}$$ (18a)

$$\Delta_{56}=\{[1.1\times 10^{-6}]-j[6{.2\times 10}^{-5}]\}$$ (18b)

for 100 pF lead capacitances and

$$\Delta_{56}=\{[2.8\times 10^{-6}]-j[6{.5\times 10}^{-5}]\}$$ (19)

for 250 pF coaxial leads.

The 1.1 × 10^−6^ in-phase Δ_56_ correction to *R*_H_for 100 pF leads is nearly two orders of magnitude larger than the 2.0 × 10^−8^ in-phase correction predicted in Ref. [16], which neglected the wire-to-wire capacitance effects. When the wire-to-wire capacitances are all reduced to 1 × 10^−14^ F, the in-phase and out-of-phase corrections are

$$\Delta_{56}=\{[1.3\times 10^{-7}]-j[8{.0\times 10}^{-6}]\}$$ (20a)

and

$$\Delta_{56}=\{[3.3\times 10^{-7}]-j[1{.1\times 10}^{-5}]\}$$ (20b)

for 100 pF and 250 pF coaxial leads, respectively.

The in-phase corrections to Δ_56_ in these examples are far greater than our desired 1 × 10^−8^ *R*_H_ absolute one-standard-deviation accuracy, making the single-series “offset” circuit *useless* for ac quantum Hall voltage measurements. However, we will see in Sec. 6.4 that this circuit *can* be used for ac longitudinal voltage measurements. We next give the approximate solutions for the currents and the quantum Hall voltage to show the source of this in-phase problem.

### 6.3 Approximate Single-Series “Offset” Solutions

The terms in the following approximate solutions were obtained in a tedious, weeks-long process by changing the individual values of circuit element components by an order of magnitude in the computer program; observing the calculated results; and then finding, by “educated guesses” and “trial-and-error”, algebraic expressions that duplicate these results. The approximate solutions yield results that agree to within at least two significant figures for both the real and imaginary parts of the exact numerical results listed in Eqs. (15) to (16) and Eqs. (18) to (20).

All the terms in the approximate equations were found to be necessary for the particular sets of circuit components values tried so far, but other terms may very well need to be added to these approximate equations if the circuit components have values significantly different from the cardinal numbers listed in Eq. (14). The reader should again be cautioned that it is the *exact* equations that are the most reliable, not the approximate equations. However, the approximate solutions provide physical insight into the sources of error in quantum Hall voltage measurements.

Let

$$C_{X^{\prime }X^{\prime }}=C_{S^{\prime }D^{\prime }}+C_{S^{\prime }1^{\prime }}+C_{S^{\prime }3^{\prime }}+C_{S^{\prime }5^{\prime }}+C_{2^{\prime }D^{\prime }}+C_{4^{\prime }D^{\prime }}+C_{6^{\prime }D^{\prime }}+C_{1^{\prime }2^{\prime }}+C_{3^{\prime }4^{\prime }}+C_{5^{\prime }6^{\prime }}.$$ (21)

Then

$$\begin{array}{c} I_{C_{S}}\approx I_{C_{S_{a}}}=\{[\omega^{2}C_{S}C_{S}r_{S}r_{S}-\omega^{2}C_{S}L_{S}-\omega^{2}C_{S}(C_{6^{\prime }D^{\prime }}+C_{5^{\prime }6^{\prime }})R_{H}R_{H}]\\ +j\left[\omega C_{S}(r_{S}+r_{d})-\omega C_{6^{\prime }D^{\prime }}\left(\frac{C_{1}+C_{3}+C_{5}}{C_{S}}\right)r_{S}\right]\}I_{\text{Ot}} \end{array}$$ (22a)

$$I_{S}\approx I_{S_{a}}=\{1-j[\omega C_{S}(r_{S}+r_{d})]\}I_{\text{Ot}}$$ (22b)

$$\begin{array}{c} I_{C_{B}}\approx I_{C_{B_{a}}}=\{[\omega^{2}C_{B}C_{S}r_{S}r_{d}-\omega^{2}C_{B}(C_{6^{\prime }D^{\prime }}+C_{5^{\prime }6^{\prime }})R_{H}R_{H}]\\ +j[\omega C_{B}r_{d}-\omega^{3}C_{B}(C_{1}+C_{3}+C_{5})C_{6^{\prime }D^{\prime }}R_{H}R_{H}R_{H}]\}I_{\text{Ot}} \end{array}$$ (22c)

$$I_{K_{B}}\approx I_{K_{B_{a}}}=\left\{\left[\frac{r_{d}}{r_{K_{B}}}-\omega^{2}(C_{1}+C_{3}+C_{5})C_{6^{\prime }D^{\prime }}R_{H}R_{H}\frac{R_{H}}{r_{K_{B}}}\right]-j\left[\omega C_{S}r_{S}\frac{r_{d}}{r_{K_{B}}}-\omega (C_{6^{\prime }D^{\prime }}+C_{5^{\prime }6^{\prime }})R_{H}\frac{R_{H}}{r_{K_{B}}}\right]\right\}I_{\text{Ot}}$$ (22d)

$$\begin{array}{c} I_{d}\approx I_{d_{a}}=I_{\text{Ot}}-I_{C_{S_{a}}}-I_{C_{B_{a}}}\\ -\{j[\omega (C_{S^{\prime }D^{\prime }}+C_{S^{\prime }1^{\prime }}+C_{S^{\prime }3^{\prime }}+C_{S^{\prime }5^{\prime }})R_{H}]\}I_{\text{Ot}} \end{array}$$ (22e)

$$I_{C_{S^{\prime }5^{\prime }}}\approx I_{C_{{S^{\prime }5^{\prime }}_{a}}}=\{[\omega^{2}C_{S^{\prime }5^{\prime }}(C_{S^{\prime }D^{\prime }}+C_{S^{\prime }1^{\prime }}+C_{S^{\prime }3^{\prime }}+C_{S^{\prime }5^{\prime }})R_{H}R_{H}]+[\omega^{2}C_{S}C_{S^{\prime }5^{\prime }}R_{H}r_{S}]+j[\omega C_{S^{\prime }5^{\prime }}R_{H}]\}I_{\text{Ot}}$$ (22f)

$$\begin{array}{c} I_{6^{\prime }}\approx I_{6_{a}^{\prime }}=\{-[\omega^{2}(C_{1}+C_{3}+C_{5})C_{6^{\prime }D^{\prime }}R_{H}R_{H}]\\ +[\omega^{2}C_{5^{\prime }6^{\prime }}(C_{S^{\prime }D^{\prime }}+C_{S^{\prime }1^{\prime }}+C_{S^{\prime }3^{\prime }}+C_{S^{\prime }5^{\prime }}+C_{6^{\prime }D^{\prime }}+C_{5^{\prime }6^{\prime }})R_{H}R_{H}]\\ +j[\omega (C_{6^{\prime }D^{\prime }}+C_{5^{\prime }6^{\prime }})R_{H}]\}I_{\text{Ot}} \end{array}$$ (22g)

$$\begin{array}{c} I_{C_{5}}\approx I_{C_{5_{a}}}=\{[\omega^{2}C_{S}C_{5}R_{H}r_{S}+\omega^{2}C_{5}C_{5}R_{H}r_{S}]\\ +[\omega^{2}C_{5}(C_{S^{\prime }D^{\prime }}+C_{S^{\prime }1^{\prime }}+C_{S^{\prime }3^{\prime }}+C_{S^{\prime }5^{\prime }}+C_{6^{\prime }D^{\prime }}+C_{5^{\prime }6^{\prime }})R_{H}R_{H}]\\ +j[\omega (C_{5}R_{H}]\}I_{\text{Ot}} \end{array}$$ (22h)

$$\begin{array}{c} I_{C_{5^{\prime }6^{\prime }}}\approx I_{C_{5^{\prime }6_{a}^{\prime }}}=\{[\omega^{2}C_{5^{\prime }6^{\prime }}(C_{S^{\prime }D^{\prime }}+C_{S^{\prime }1^{\prime }}+C_{S^{\prime }3^{\prime }}+C_{S^{\prime }5^{\prime }}+C_{6^{\prime }D^{\prime }}+C_{5^{\prime }6^{\prime }})R_{H}R_{H}]\\ +[\omega^{2}C_{S}C_{5^{\prime }6^{\prime }}R_{H}r_{S}]+j[\omega C_{5^{\prime }6^{\prime }}R_{H}]\}I_{\text{Ot}} \end{array}$$ (22i)

$$I_{5^{\prime }}\approx I_{5_{a}^{\prime }}=I_{C_{5_{a}}}+I_{C_{S^{\prime }5_{a}^{\prime }}}+I_{C_{5^{\prime }6_{a}^{\prime }}}$$ (22j)

$$I_{c}\approx I_{c_{a}}=I_{d_{a}}+I_{5_{a}^{\prime }}-I_{6_{a}^{\prime }}$$ (22k)

$$I_{C_{6^{\prime }D^{\prime }}}\approx I_{C_{6^{\prime }D_{a}^{\prime }}}=I_{6_{a}^{\prime }}-I_{C_{5^{\prime }6_{a}^{\prime }}}$$ (22l)

$$\begin{array}{c} I_{C_{4}}\approx I_{C_{4_{a}}}=\{[\omega^{2}C_{4}C_{4}(R_{H}+r_{4})r_{c}-\omega^{2}C_{4}C_{5}R_{H}r_{c}]\\ +[\omega^{2}C_{S}C_{4}r_{S}r_{c}-\omega^{2}C_{4}(C_{4^{\prime }D^{\prime }}+C_{3^{\prime }4^{\prime }})R_{H}R_{H}]\\ +j[\omega C_{4}r_{c}-\omega^{3}C_{5}(C_{1}+C_{3})C_{4^{\prime }D^{\prime }}R_{H}R_{H}R_{H}]\}I_{\text{Ot}} \end{array}$$ (22m)

$$\begin{array}{c} I_{4^{\prime }}\approx I_{4_{a}^{\prime }}=-I_{C_{4_{a}}}+\{-[\omega^{2}(C_{1}+C_{3}+C_{5})C_{4^{\prime }D^{\prime }}R_{H}R_{H}\\ +\omega^{2}C_{5}C_{3^{\prime }4^{\prime }}R_{H}R_{H}]+[\omega^{2}C_{3^{\prime }4^{\prime }}(C_{X^{\prime }X^{\prime }}-C_{S^{\prime }5^{\prime }}-C_{5^{\prime }6^{\prime }})R_{H}R_{H}]\\ +j[\omega (C_{4^{\prime }D^{\prime }}+C_{3^{\prime }4^{\prime }})R_{H}]\}I_{\text{Ot}} \end{array}$$ (22n)

$$\begin{array}{c} I_{C_{3}}\approx I_{C_{3_{a}}}=\{-[\omega^{2}C_{3}C_{5}R_{H}R_{H}]\\ +[\omega^{2}C_{3}(C_{S^{\prime }D^{\prime }}+C_{S^{\prime }1^{\prime }}C_{S^{\prime }3^{\prime }}+C_{6^{\prime }D^{\prime }})R_{H}R_{H}]\\ +j[\omega C_{3}R_{H}]\}I_{\text{Ot}} \end{array}$$ (22o)

$$\begin{array}{c} I_{C_{S^{\prime }3^{\prime }}}\approx I_{C_{{S^{\prime }3^{\prime }}_{a}}}=\{-[\omega^{2}C_{5}C_{S^{\prime }3^{\prime }}R_{H}R_{H}]\\ +[\omega^{2}C_{S^{\prime }3^{\prime }}(C_{S^{\prime }D^{\prime }}+C_{S^{\prime }1^{\prime }}C_{S^{\prime }3^{\prime }})R_{H}R_{H}]\\ +j[\omega C_{S^{\prime }3^{\prime }}R_{H}]\}I_{\text{Ot}} \end{array}$$ (22p)

$$\begin{array}{c} I_{C_{3^{\prime }4^{\prime }}}=I_{C_{3^{\prime }4_{a}^{\prime }}}=\{-[\omega^{2}C_{5}C_{3^{\prime }4^{\prime }}R_{H}R_{H}]\\ +[\omega^{2}C_{3^{\prime }4^{\prime }}(C_{S^{\prime }D^{\prime }}+C_{S^{\prime }1^{\prime }}+C_{S^{\prime }3^{\prime }}+C_{6^{\prime }D^{\prime }}+C_{4^{\prime }D^{\prime }}+C_{3^{\prime }4^{\prime }})R_{H}R_{H}]\\ +j[\omega C_{3^{\prime }4^{\prime }}R_{H}]\}I_{\text{Ot}} \end{array}$$ (22q)

$$I_{3^{\prime }}\approx I_{3_{a}^{\prime }}=I_{C_{3_{a}}}+I_{C_{{S^{\prime }3^{\prime }}_{a}}}+I_{C_{{3^{\prime }4^{\prime }}_{a}}}$$ (22r)

$$I_{b}\approx I_{b_{a}}=I_{\text{Ot}}+I_{C_{5_{a}}}+I_{C_{3_{a}}}$$ (22s)

$$\begin{array}{c} I_{C_{2}}\approx I_{C_{2_{a}}}=\{-[\omega^{2}C_{2}(C_{4^{\prime }D^{\prime }}+C_{2^{\prime }D^{\prime }}+C_{1^{\prime }2^{\prime }}+C_{3^{\prime }4^{\prime }})R_{H}R_{H}]\\ -[\omega^{2}C_{2}C_{3}R_{H}r_{b}-\omega^{2}C_{2}C_{4}R_{H}r_{c}-\omega^{2}C_{2}C_{S^{\prime }3^{\prime }}R_{H}r_{c}]\\ +[\omega^{2}C_{2}(C_{2}-C_{5})R_{H}(r_{b}+r_{c})+\omega^{2}C_{3}C_{4}r_{2}r_{c}]\\ +[\omega^{2}C_{S}C_{2}r_{S}(r_{b}+r_{c})]+j[\omega C_{2}(r_{b}+r_{c})]\\ -j[\omega^{3}C_{2}C_{5}(C_{4^{\prime }D^{\prime }}+C_{2^{\prime }D^{\prime }}+C_{1^{\prime }2^{\prime }}+C_{3^{\prime }4^{\prime }})R_{H}R_{H}R_{H}]\\ +j[\omega^{3}C_{2}(C_{2}+C_{4})C_{3^{\prime }4^{\prime }})R_{H}R_{H}R_{H}]\\ -j[\omega^{3}C_{2}C_{3}C_{2^{\prime }D^{\prime }}R_{H}R_{H}R_{H}]\}I_{\text{Ot}} \end{array}$$ (22t)

$$\begin{array}{c} I_{2^{\prime }}\approx I_{2_{a}^{\prime }}=-I_{C_{2_{a}}}+\{-\omega^{2}(C_{1}+C_{3}+C_{5})C_{2^{\prime }D^{\prime }}R_{H}R_{H}]\\ -[\omega^{2}(C_{3}+C_{5})C_{1^{\prime }2^{\prime }}R_{H}R_{H}]\\ +j[\omega (C_{2^{\prime }D^{\prime }}+C_{1^{\prime }2^{\prime }})R_{H}]\}I_{\text{Ot}} \end{array}$$ (22u)

$$I_{C_{1}}\approx I_{C_{1_{a}}}=\{-[\omega^{2}C_{1}(C_{3}+C_{5})R_{H}R_{H}]+j[\omega C_{1}R_{H}]\}I_{\text{Ot}}$$ (22v)

$$I_{C_{S^{\prime }1^{\prime }}}\approx I_{C_{S^{\prime }1_{a}^{\prime }}}=\{-[\omega^{2}C_{S^{\prime }1^{\prime }}(C_{3}+C_{5})R_{H}R_{H}]+j[\omega C_{S^{\prime }1^{\prime }}R_{H}]\}I_{\text{Ot}}$$ (22w)

$$I_{C_{1^{\prime }2^{\prime }}}\approx I_{C_{1^{\prime }2_{a}^{\prime }}}=\{-[\omega^{2}C_{1^{\prime }2^{\prime }}(C_{3}+C_{5})R_{H}R_{H}]+j[\omega C_{1^{\prime }2^{\prime }}R_{H}]\}I_{\text{Ot}}$$ (22x)

$$I_{1^{\prime }}\approx I_{1_{a}^{\prime }}=I_{C_{1_{a}}}+I_{C_{{S^{\prime }1^{\prime }}_{a}}}+I_{C_{{S^{\prime }2^{\prime }}_{a}}}$$ (22y)

$$I_{a}\approx I_{a_{a}}=I_{\text{Ot}}+I_{C_{5_{a}}}+I_{C_{3_{a}}}+I_{C_{1_{a}}}$$ (22z)

$$\begin{array}{c} I_{C_{4^{\prime }D^{\prime }}}=I_{C_{4^{\prime }D_{a}^{\prime }}}=\{-[\omega^{2}C_{4^{\prime }D^{\prime }}(C_{1}+C_{3}+C_{5})R_{H}R_{H}]\\ +j[\omega C_{4^{\prime }D^{\prime }}R_{H}]\}I_{\text{Ot}} \end{array}$$ (23a)

$$\begin{array}{c} I_{C_{2^{\prime }D^{\prime }}}=I_{C_{2^{\prime }D_{a}^{\prime }}}=\{-[\omega^{2}C_{2^{\prime }D^{\prime }}(C_{1}+C_{3}+C_{5})R_{H}R_{H}]\\ +j[\omega C_{2^{\prime }D^{\prime }}R_{H}]\}I_{\text{Ot}} \end{array}$$ (23b)

$$\begin{array}{c} I_{C_{S^{\prime }D^{\prime }}}\approx I_{C_{{S^{\prime }D^{\prime }}_{a}}}=\{-[\omega^{2}C_{S^{\prime }D^{\prime }}(C_{1}+C_{3}+C_{5})R_{H}R_{H}]\\ +j[\omega C_{S^{\prime }D^{\prime }}R_{H}]\}I_{\text{Ot}} \end{array}$$ (23c)

$$I_{K_{A}}\approx I_{K_{A_{a}}}=\left\{\left[\frac{R_{H}}{r_{K_{A}}}\right]+j\left[\omega (C_{1}+C_{3}+C_{5})R_{H}\frac{R_{H}}{r_{K_{A}}}\right]\right\}I_{\text{Ot}}$$ (23d)

$$I_{C_{A}}\approx I_{C_{A_{a}}}=\{-[\omega^{2}C_{A}(C_{1}+C_{3}+C_{5})R_{H}R_{H}]+j[\omega C_{A}R_{H}]\}I_{\text{Ot}}$$ (23e)

$$I_{D}\approx I_{D_{a}}=I_{\text{Ot}}+I_{C_{5_{a}}}+I_{C_{3_{a}}}++I_{C_{1_{a}}}+I_{C_{A_{a}}}$$ (23f)

$$I_{C_{D}}\approx I_{C_{D_{a}}}=\{-[\omega^{2}C_{D}(C_{1}+C_{3}+C_{5})R_{H}R_{H}]+j[\omega C_{D}R_{H}]]\}I_{\text{Ot}}$$ (23g)

$$I_{\text{Dr}}\approx I_{{\text{Dr}}_{a}}=I_{\text{Ot}}+I_{C_{5_{a}}}+I_{C_{3_{a}}}+I_{C_{1_{a}}}+I_{C_{A_{a}}}+I_{C_{D_{a}}}.$$ (23h)

Expressing Eq. (17a) in the form (17b),

$$\begin{array}{c} \Delta_{56}\approx \Delta_{56_{a}}=\{-[\omega^{2}C_{B}C_{5}R_{H}r_{c}+\omega^{2}C_{S}C_{S}r_{S}r_{S}+\omega^{2}C_{S}C_{5}r_{S}r_{5}]\\ -[\omega^{2}C_{5}C_{5}r_{5}r_{5}-\omega^{2}C_{S}L_{S}-\omega^{2}C_{5}L_{5}]\\ +[\omega^{2}C_{S}(C_{6^{\prime }D^{\prime }}+C_{5^{\prime }6^{\prime }})R_{H}R_{H}]\\ +[\omega^{2}C_{1}(C_{S^{\prime }D^{\prime }}+C_{6^{\prime }D^{\prime }})R_{H}R_{H}]\\ +[\omega^{2}C_{3}(C_{S^{\prime }D^{\prime }}+C_{S^{\prime }1^{\prime }}+C_{6^{\prime }D^{\prime }})R_{H}R_{H}]\\ +[\omega^{2}C_{5}(C_{S^{\prime }D^{\prime }}+C_{S^{\prime }1^{\prime }}+C_{S^{\prime }3^{\prime }}+C_{6^{\prime }D^{\prime }})R_{H}R_{H}]\\ -j[\omega C_{S}r_{S}+\omega C_{5}r_{5}+\omega C_{B}(r_{c}+r_{d})]\\ -j[\omega (C_{S^{\prime }D^{\prime }}+C_{S^{\prime }1^{\prime }}+C_{S^{\prime }3^{\prime }}+C_{S^{\prime }5^{\prime }}+C_{6^{\prime }D^{\prime }}+C_{5^{\prime }6^{\prime }})R_{H}]\}. \end{array}$$ (24)

It would have been very difficult to predict some of these approximate current and voltage solutions without first knowing the exact results. We see from Eq. (24) that the main source of the intolerably large in-phase corrections to the quantum Hall voltage signal arises from terms involving the products of capacitances-to-shield times wire-to-wire capacitances.

Although it is disappointing that single-series “offset” connections cannot be used to make ac quantum Hall voltage measurements, we see in the next subsection that the circuit analysis remains applicable for ac longitudinal voltage measurements, and that this circuit *can* be used for ac longitudinal voltage measurements.

### 6.4 AC Longitudinal Voltage Measurements

**Fig. 3:**
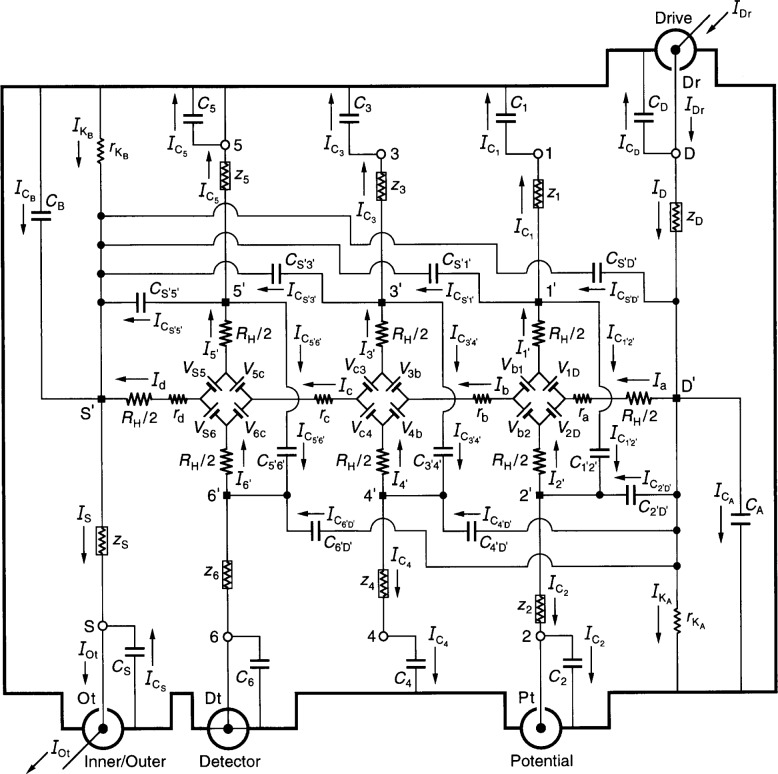
An equivalent electrical circuit representation of an ac QHE resistance standard while being measured for the longitudinal voltage *V_x_*(2,6) using four-terminal-pair techniques. See Sec. 6.4 for the circuit analysis.

The Potential port shown in Fig. 3 has been moved from room temperature access point 5 in Fig. 2 to room temperature access point 2. Now the ac longitudinal voltage *V_x_*(2,6) is measured, rather than the ac quantum Hall voltage *V*_H_(5,6). Also, the ac longitudinal voltage *V_x_*(4,6) can be measured by simply moving the Potential port from access point 2 in Fig. 3 to point 4.

Notice that the four-terminal-pair definitions given in Sec. 5 still apply since *I*_Pt_ = 0 and *I*_Dt_ = 0 for both the *V_x_*(2,6) and *V_x_*(4,6) measurements. The equivalent circuit element currents in Fig. 3 are *identical* to those shown in Fig. 2. Therefore, the exact currents derived in Sec. 6.1; the numerical values listed in Sec. 6.2; and the approximate currents given by Eqs. (22) and (23) in Sec. 6.3 remain unchanged.

The exact equation for the ac longitudinal voltage *V_x_*(4,6) is obtained by summing the voltages between the inner conductors of the Detector coaxial port and the Potential coaxial port. Taking the path through arm 6, voltage generators *V*_6c_ and *V*_c4_, and arm 4 we find that

$$V_{x}(4,6)=r_{c}I_{c}+R_{H}I_{4^{\prime }}-z_{4}I_{C_{4}},$$ (25a)

which can also be expressed in the form

$$V_{x}(4,6)=[1+\Delta_{46}]r_{c}I_{\text{Ot}}.$$ (25b)

Equation (25b) can be rewritten as

$$V_{x}(4,6)-r_{c}I_{\text{Ot}}=\Delta_{46}r_{c}I_{\text{Ot}}=\delta_{46}R_{H}I_{\text{Ot}}.$$ (25c)

Thus

$$\Delta_{46}=\frac{\left[V_{x}(4,6)-r_{c}I_{\text{Ot}}\right]}{r_{c}I_{\text{Ot}}},$$ (25d)

and

$$\delta_{46}=\frac{r_{c}}{R_{H}}\Delta_{46}.$$ (25e)

Likewise,

$$V_{x}(2,6)=r_{c}I_{c}+r_{b}I_{b}+R_{H}I_{4^{\prime }}+R_{H}I_{2^{\prime }}-z_{2}I_{C_{2}},$$ (26a)

which can be expressed in the form

$$V_{x}(2,6)=[1+\Delta_{26}](r_{c}+r_{b})I_{\text{Ot}}.$$ (26b)

Equation (26b) can be rewritten as

$$V_{x}(2,6)-(r_{c}+r_{b})I_{\text{Ot}}=\Delta_{26}(r_{c}+r_{b})I_{\text{Ot}}=\delta_{26}R_{H}I_{\text{Ot}}.$$ (26c)

Thus

$$\Delta_{26}=\frac{\left[V_{x}(2,6)-(r_{c}+r_{b})I_{\text{Ot}}\right]}{(r_{c}+r_{b})I_{\text{Ot}}},$$ (26d)

and

$$\delta_{26}=\frac{\left(r_{c}+r_{b}\right)}{R_{H}}\Delta_{26}.$$ (26e)

The numerical results of Eq. (25), when using the cardinal values listed in Eq. (14), are

$$V_{x}(4,6)=\{1-[1.99160]+j[199.990]\}r_{c}I_{\text{Ot}}$$ (27a)

$$\Delta_{46}=\{-[1.99160]+j[199.990]\}$$ (27b)

$$\delta_{46}=\{-[2.0\times 10^{-7}]+j[2.0\times 10^{-5}]\}$$ (27c)

for 100 pF lead capacitances and

$$\Delta_{46}=\{-[4.99103]+j[199.938]\}$$ (28a)

$$\delta_{46}=\{-[5.0\times 10^{-7}]+j[2.0\times 10^{-5}]\}$$ (28b)

for 250 pF coaxial leads.

The numerical results of Eq. (26), when using the cardinal values listed in Eq. (14), are

$$V_{x}(2,6)=\{1-[1.49070]+j[199.990]\}(r_{c}+r_{b})I_{\text{Ot}}$$ (29a)

$$\Delta_{26}=\{-[1.49070]+j[199.990]\}$$ (29b)

$$\delta_{26}=\{-[3.0\times 10^{-7}]+j[4.0\times 10^{-5}]\}$$ (29c)

for 100 pF lead capacitances and

$$\Delta_{26}=\{-[3.74112]+j[199.938]\}$$ (30a)

$$\delta_{26}=\{-[7.5\times 10^{-7}]+j[4.0\times 10^{-5}]\}$$ (30b)

for 250 pF coaxial leads.

We see from Eqs. (27b) and (28a) that the measured ac values of *V_x_*(4,6) have in-phase errors of − 2*r*_c_*I*_Ot_ for 100 pF leads and − 5 *r*_c_*I*_Ot_ for 250 pF leads. These errors are due to the parasitic impedances. The corresponding errors for *V_x_*(2,6) measurements are − 1.49(*r*_c_+ *r*_b_)*I*_Ot_ and − 3.74(*r*_c_+ *r*_b_)*I*_Ot_in Eqs. (29b) and (30a), respectively. The − 5 *r*_c_in-phase error in measuring the ac value of *r*_c_for 250 pF leads is too large to apply a reasonable correction. This error is reduced to a more tolerable − 2 *r*_c_in-phase error with shorter, 100 pF, sample probes. Furthermore, the error signal in Eq. (27b) is reduced by a factor of ten to Δ_46_ = {−[0.19988] + j[19.9990} when the wire-to-wire capacitances are lowered from our assumed 10^−+×^ F values to 10^−14^ F values. It is thus very important to reduce the capacitances-to-shield and the wire-to-wire capacitances as much as possible when making ac longitudinal voltage measurements.

The numerical examples in Eqs. (27) through (30) have assumed 1 mΩ ac longitudinal resistance values for *r*_a_, *r*_b_, *r*_c_, and *r*_d_. Equations (27b) and (27c) become

$$\Delta_{46}=\{-[0.20000]+j[20.0000]\}$$ (31a)

$$\delta_{46}=\{-[2.0\times 10^{-7}]+j[2.0\times 10^{-5}]\}$$ (31b)

for 100 pF leads and 10 mΩ longitudinal ac resistances and

$$\Delta_{46}=\{-[20.0000]+j[2000.00]\}$$ (32a)

$$\delta_{46}=\{-[2.0\times 10^{-7}]+j[2.0\times 10^{-5}]\}$$ (32b)

for 100 pF leads and 0.1 mΩ longitudinal resistances. Equations (27b), (31a), and (32a) demonstrate that the contributions from the parasitic impedances to the in-phase components of the ac longitudinal resistance errors, Δ_46_*r*_c_, remain unchanged when the values of *r*_a_, *r*_b_, *r*_c_, and *r*_d_vary. Equations (27c), (31b), and (32b) demonstrate that the ac longitudinal resistance errors, δ_46_, (which are expressed as fractions of the quantized Hall resistance *R*_H_) are *independent* of the values of *r*_a_, *r*_b_, *r*_c_, and *r*_d_.

There are very large out-of-phase errors for all the examples given by Eqs. (27) through (32). We will see from the approximate equations listed below in Eqs. (33) and (34) that these out-of-phase errors, as well as the in-phase errors, arise from the parasitic impedances.

The out-of-phase component of the ac longitudinal voltage signal, such as the {j[199.990} *r*_c_*I*_Ot_ term of Eq. (27a), is nulled with bridge balances. That is normally not a problem because impedance standards usually have small capacitive components if they are ac resistors, or small resistances if they are capacitors. However, ac quantized Hall resistance standards have large out-of-phase components of the ac longitudinal voltage signal due to the parasitic impedances. For example, the out-of-phase signal term is {j[2.0 × 10^−^=}*R*_H_*I*_Ot_in Eq. (27c) when expressed as a fraction of *R*_H_. Balances in NIST high precision ac bridges are capable of providing out-of-phase adjustment signals as large as 5 × 10^−4^ *R*_H_*I*_Ot_, so the bridges can easily null this out-of-phase signal, but the following error can occur. The components used in the bridge balances to null the out-of-phase component of the ac longitudinal voltage signal have resistive, in-phase (phase defect) contributions, so when we inject an out-of phase signal to null the out-of-phase ac longitudinal voltage signal we inadvertently also inject a small, real, in-phase component. The in-phase (phase defect) balance signal is unintentionally *added* to the real in-phase component of the ac longitudinal voltage signal. Therefore, the bridge must be carefully calibrated so that we know precisely how much in-phase signal has been inadvertently injected, and can apply corrections for this injected signal.

An uncorrected phase defect error in the measured longitudinal voltage has a *linear* frequency dependence in NIST four-terminal-pair bridges. This error may be a possible source of the linear frequency dependences of the ac longitudinal voltages reported by other laboratories.

The ac longitudinal voltage can also be measured by *not* nulling the in-phase and the large out-of-phase voltage signals at the lock-in detector, and instead calibrating the detector linearity and then using the direct (unbalanced) in-phase and out-of-phase detector readings. The lock-in detector would require separate sensitivity ranges for the in-phase and the out-of-phase components of the voltage signal since these components differ by two orders of magnitude in our numerical examples. This direct reading method would be a useful check of the ac longitudinal voltage measurements made by balancing the bridge and nulling the lock-in detector.

These difficult problems of measuring the ac longitudinal voltages and then correcting for parasitic impedance contributions and bridge balance effects or lock-in detector nonlinearities disappear if it can ultimately be experimentally shown that there are no intrinsic frequency dependences of the QHE devices themselves. Then dc values of *r*_a_, *r*_b_, *r*_c_, and *r*_d_can be assigned to Fig. 3, and to the exact current and longitudinal voltage equations, by using dc measurements under the same sample temperature conditions as in the ac measurements, and during the same cool-down.

The terms in the following approximate corrections to the ac longitudinal voltage were again obtained by changing the individual values of circuit element components by an order of magnitude in the computer software; observing the calculated results; and then finding, by “educated guesses” and “trial-and-error”, algebraic expressions that duplicate these results. The approximate corrections yield results that agree to within at least two significant figures for both the real and imaginary parts of the exact numerical results listed in Eqs. (27) to (32). All the terms in the approximate corrections were found to be necessary for the particular sets of circuit components values tried so far, but other terms may need to be added to these approximate corrections if the circuit components have values significantly different from the cardinal numbers listed in Eq. (14). The reader should again be cautioned that it is the *exact* equations given by Eqs. (25) and (26) that are the most reliable, not the approximate equations. However, the approximate equations provide physical insight into the sources of error in ac longitudinal voltage measurements.

The approximate corrections to the ac longitudinal voltage are expressed as fractions of the quantized Hall resistance *R*_H_. They can also be expressed in terms of the longitudinal resistances *r*_c_or (*r*_c_+ *r*_b_) by multiplying each term in Eq. (33) by the quantity *R*_H_/*r*_c_, and each term in Eq. (34) by the quantity *R*_H_/(*r*_c_+ *r*_b_). The approximate corrections to the ac longitudinal voltages are

$$\begin{array}{l} \delta_{46}\approx \delta_{46}=\{-[\omega^{2}C_{4}C_{4}(R_{H}+r_{4})r_{c}-\omega^{2}C_{4}C_{5}R_{H}r_{c}]\\ -[\omega^{2}C_{S}(C_{4}-C_{5})r_{S}r_{c}-\omega^{2}C_{4}C_{5}r_{4}r_{c}-\omega^{2}C_{5}C_{5}r_{5}r_{c}]\\ -\left[\omega^{2}C_{4}C_{4}\left(\frac{R_{H}+r_{2}}{R_{H}}\right)r_{4}r_{c}+\omega^{2}C_{S}C_{S}\left(\frac{r_{S}}{R_{H}}\right)r_{S}r_{c}\right]\\ -\left[\omega^{2}C_{S}C_{4}\left(\frac{r_{S}}{R_{H}}\right)r_{4}r_{c}-\omega^{2}C_{S}L_{S}\left(\frac{r_{c}}{R_{H}}\right)\right]\\ -[\omega^{2}(C_{1}+C_{3})C_{4^{\prime }D^{\prime }}R_{H}R_{H}]\\ -j\left[\omega \left(C_{4}-C_{5}\right)r_{c}+\omega C_{S}\left(\frac{r_{S}+r_{d}}{R_{H}}\right)r_{c}+\omega C_{4}\left(\frac{r_{4}}{R_{H}}\right)r_{c}\right]\\ \;+j[\omega (C_{4^{\prime }D^{\prime }}+C_{3^{\prime }4^{\prime }})R_{H}]\} \end{array}$$ (33)

and

$$\begin{array}{l} \delta_{26}\approx \delta_{26_{a}}=\{-[\omega^{2}C_{4}C_{4}(R_{H}+r_{4})r_{c}-\omega^{2}C_{4}C_{5}R_{H}r_{c}]\\ -[\omega^{2}C_{2}C_{4}R_{H}r_{c}-\omega^{2}C_{3}(C_{2}-C_{5})R_{H}r_{b}]\\ -[\omega^{2}C_{2}(C_{2}-C_{5})R_{H}(r_{c}+r_{b})+\omega^{2}C_{3}C_{4}r_{2}r_{c}]\\ -[\omega^{2}C_{S}C_{4}r_{S}r_{c}-\omega^{2}C_{S}C_{5}r_{S}(r_{c}+r_{b})]\\ +[\omega^{2}C_{5}C_{5}r_{5}(r_{c}+r_{b})-\omega^{2}C_{S}C_{2}r_{S}(r_{c}+r_{b})]\\ -\left[\omega^{2}C_{S}C_{S}\left(\frac{r_{S}}{R_{H}}\right)r_{S}\left(r_{c}+r_{b}\right)-\omega^{2}C_{S}L_{S}\left(\frac{r_{c}+r_{b}}{R_{H}}\right)\right]\\ -[\omega^{2}C_{1}(C_{2^{\prime }D^{\prime }}+C_{4^{\prime }D^{\prime }})R_{H}R_{H}]\\ +[\omega^{2}C_{2}(C_{2^{\prime }D^{\prime }}+C_{4^{\prime }D^{\prime }}+C_{1^{\prime }2^{\prime }}+C_{3^{\prime }4^{\prime }})R_{H}R_{H}]\\ -[\omega^{2}C_{3}(C_{2^{\prime }D^{\prime }}+C_{4^{\prime }D^{\prime }}+C_{1^{\prime }2^{\prime }})R_{H}R_{H}]\\ +[\omega^{2}C_{4}(C_{4^{\prime }D^{\prime }}+C_{3^{\prime }4^{\prime }})R_{H}R_{H}]\\ -[\omega^{2}C_{5}(C_{2^{\prime }D^{\prime }}+C_{4^{\prime }D^{\prime }}+C_{1^{\prime }2^{\prime }}+C_{3^{\prime }4^{\prime }})R_{H}R_{H}]\\ -j[\omega C_{2}(r_{c}+r_{b})-\omega C_{3}r_{b}+\omega C_{4}r_{c}-\omega C_{5}(r_{c}+r_{b})]\\ -j\left[\omega C_{S}\left(\frac{r_{S}}{R_{H}}\right)\left(r_{c}+r_{b}\right)+\omega C_{S}\left(\frac{r_{d}}{R_{H}}\right)\left(r_{c}+r_{b}\right)\right]\\ -j\left[\omega C_{2}\left(\frac{r_{2}}{R_{H}}\right)\left(r_{c}+r_{b}\right)\right]\\ \;+j[\omega (C_{2^{\prime }D^{\prime }}+C_{4^{\prime }D^{\prime }}+C_{1^{\prime }2^{\prime }}+C_{3^{\prime }4^{\prime }})R_{H}]\}. \end{array}$$ (34)

We see from Eqs. (33) and (34) that the main sources of the in-phase corrections to the ac longitudinal voltage signal arise from terms involving the products of capacitances-to-shield times wire-to-wire capacitances. The main sources of the large out-of-phase corrections to the ac longitudinal voltage signal arise from terms involving the wire-to-wire capacitances.

## 7. Analysis of the Quadruple-Series Circuit

**Fig. 4:**
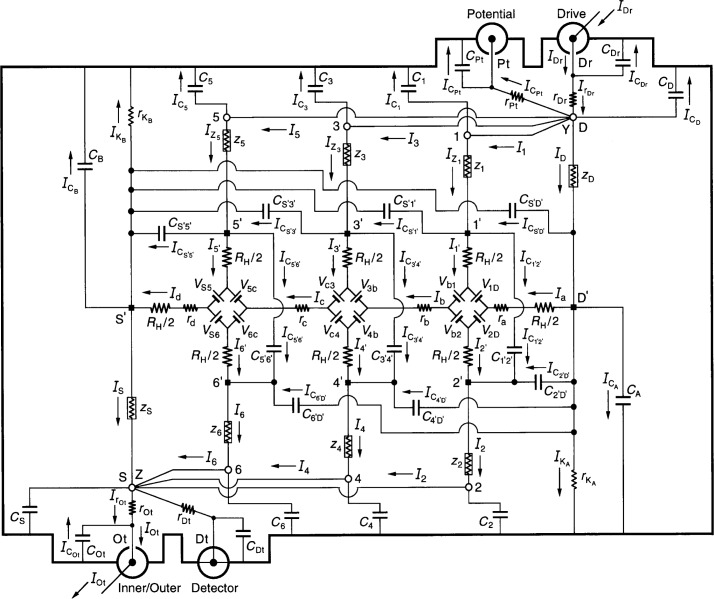
An equivalent electrical circuit representation of an ac QHE resistance standard with two quadruple-series connections to the device. See Sec. 7 for the circuit analysis.

Figure 4 shows an equivalent electrical circuit representation of an ac QHRS using two *quadruple-series* connections to the QHE device. There are *two* quadruple-series connections because *one* set of four short coaxial leads, located outside the sample probe, connect room temperature access points 5, 3, 1, and D at point Y, providing four current paths to the device. *Another* set of four short coaxial leads connect access points 2, 4, 6, and S at point Z. Short coaxial leads, also located outside the sample probe, connect point Y with the Drive and Potential ports, and point Z with the Inner/Outer and Detector ports.

This quadruple-series circuit is considered here because it provided the desired accuracy in earlier stages of the analysis [13,16] with all sample probe leads attached. All other circuit connection possibilities (except the single-series “offset” circuit examined in Sec. 6) had been eliminated in Refs. [13] and [16] due to undesirable parasitic impedance effects.

### 7.1 Exact Quadruple-Series Equations

We again use Kirchoff’s rules to sum the voltages around loops and the currents at branch points to obtain exact algebraic equations for the equivalent electrical circuit shown in Fig. 4. Eight of the current solutions are trivial because of the four-terminal-pair definition [23,24] listed in Sec. 5

$$I_{\text{Dt}}=I_{\text{Pt}}=I_{C_{S}}=I_{C_{\text{Dt}}}=I_{C_{2}}=I_{C_{4}}=I_{C_{6}}=I_{\text{rDt}}=0.$$ (35)

Those eight currents are not included in the 46 inner conductor currents shown in the figure since they are zero in the ideal four-terminal-pair definition. (They can be adjusted to be zero within several parts in 10^9^ of *I*_Ot_ in NIST bridges.) The remaining circuit has 27 independent voltage loop equations and 18 independent current branch points. This gives a set of 45 coupled equations for the 46 non-zero currents. Our goal, once again, is to simultaneously solve this set of 45 coupled equations, and to then express all the currents, and the quantum Hall voltage, in terms of *I*_Ot_ because that is the current which enters the ac reference standard (not shown in Fig. 4).

At bridge balance, the measured ac quantized Hall voltage for this circuit is defined as

$$V_{\text{Pt}}=V_{H}\left(Y,Z\right)-r_{\text{Pt}}I_{C_{\text{Pt}}}.$$ (36)

The voltage *V*_H_(Y,Z) between room temperature access points Y and Z can be expressed as

$$V_{H}\left(Y,Z\right)=[1+\Delta_{\text{YZ}}]R_{H}\left(i\right)I_{\text{Ot}},$$ (37)

where Δ_YZ_is the correction factor to the intrinsic quantized Hall resistance of the QHE device, *R*_H_*(i)*. This correction factor Δ_YZ_is to be determined in the analysis.

We again assume that *R*_H_*(i)* is a function of temperature and current, and it can therefore differ from the ideal value *h*/(*e*^2^*i*). Measured values of *V*_Pt_also vary with frequency [4–9]. We assume: (a) that this frequency dependence arises from the parasitic impedance effects of the ac quantized Hall resistance standard, as represented by the correction factor Δ_YZ_; (b) that the intrinsic quantized Hall resistance *R*_H_*(i)* is not frequency dependent; and (c) that the dc values are appropriate for the *R*_H_*(i)*/2 resistances in Fig. 4. (The last assumption does not preclude adding a frequency dependence to the measured dc values of *R*_H_*(i)* if the predicted value of *V*_H_(Y,Z) does not agree with the measured value.)

Finding the exact algebraic equations for all the currents, and for the correction factor Δ_YZ_to the quantum Hall voltage as defined by Eq. (37), is difficult because of the many coupled equations. However, it is important to obtain the *exact* solutions, rather than initially guess approximate solutions, because the frequency dependent effects we are trying to minimize or eliminate are small, but significant.

The algebraic derivations were again done “by hand”, rather than with computer software programs, because doing it “by hand” allowed us to investigate each stage of the solution, and to get a physical sense of the equations. Computer software programs generated pages of equations, with no simplification and no physical insight. Computer programs were, however, again used to calculate numerical examples. We used the procedures described in the next two paragraphs to find the exact solutions.

Two of the authors independently derived the equations. They made the same choices of current branch points, but different choices of paths for the independent voltage loops and substitutions of algebraic variables. As described above, there are 45 coupled equations and 46 non-zero currents for this circuit. However, 5 of the currents ( $I_{C_{\text{Pt}}}$, $I_{C_{D}}$, $I_{r_{\text{Dr}}}$, $I_{C_{\text{Dr}}}$, and *I*_Dr_) can be easily obtained after the other currents are individually expressed as functions of *I*_Ot_. For example, $I_{C_{D}}$ can be obtained once *I*_D_and $I_{C_{A}}$ are known by using the voltage loop *C*_A_, *z*_D_, *C*_D_, through the shield, and back to *C*_A_. The crux of the problem is to solve the remaining 40 branch and loop equations containing 41 coupled currents.

The two authors used different strategies to “decouple” these 40 initial equations and thereby derive the results. They then *independently* used Mathcad PLUS 6 (Professional Edition for Macintosh computers)^1^ software to enter their *own* algebraic substitutions and their *own* current equations, and then compared numerical results. The 92 in-phase and out-of-phase components of the 46 currents were in agreement to within at least six significant figures for 80 of the components when using the highest allowed precision of the software and when the values were expressed in the scientific notation 1.23456 × 10^−yz^. The results agreed to within five significant figures for three other components, four significant figures for one component, three significant figures for another component, and two significant figures for yet another component. Therefore, almost all results were in good to excellent agreement. However, six components disagreed in the first significant figure. These six components were the in-phase and out-of-phase (imaginary) terms of *I*_1′_ and *I*_3′_, and the in-phase terms of $I_{z_{1}}$ and $I_{z_{3}}$. A troubling aspect of this disagreement was that one author showed that the numerical values of the in-phase term of $I_{z_{1}}$ for that solution depended on the *order* that the algebraic substitutions were calculated in the exact equation, and how the substitution terms were grouped in the equations. This suggests a cumulative progression of computer round-off errors in the calculations that affects some current components.

Both authors also calculated the case where the wire-to-wire capacitances approach zero by again using the limiting values 10^−60^ F. The results were in good to excellent agreement except for the out-of-phase term of *I*_a_ (which differed in the second significant figure) and the in-phase terms of *I*_1′_ and $I_{z_{1}}$ and out-of-phase terms of *I*_4′_ and $I_{z_{1}}$ (which differed in the first significant figure). Except for a difference in the second significant figure for the in-phase term of *I*_3′_ and in the first significant figure for the in-phase term of *I*_1′_, the exact algebraic solutions that were finally chosen and discussed below are in excellent agreement with the numerical results listed in Eqs. (48) and (49) of Ref. [16] for zero wire-to-wire capacitances.

The third author used the initial set of 40 coupled current equations discussed below, again entered those equations as a matrix array in the Mathcad^1^ software, and for particular numerical examples, inverted the matrix to obtain numerical results that were in complete agreement for almost all of the currents, and to help resolve the above discrepancies in the other currents. This method for solving the coupled equations is faster, but less reliable, than solving the equations by hand, since round-off errors can go undetected. It was not possible to extend the matrix inversion calculations to the limit of negligible wire-to-wire capacitances, but most of the other results were in good to excellent agreement with the exact solutions discussed below. However, there were differences in the second significant figure for the in-phase terms of *I*_3′_ and $I_{z_{3}}$, and in the first significant figure for both the in-phase and out-of-phase terms of *I*_1′_ and *I*_3′_.

Neither of the two exact solutions, nor the matrix inversion solution, gave a complete set of correct current equations. Each solution had several obvious inconsistencies. This is possibly due to the cumulative effects of computer round-off errors. We chose the exact solution with the fewest inconsistencies, and then eliminated those inconsistencies by substituting the approximate solutions listed in Eqs. (53u), (53w), and (54e) for the exact equations for *I*_3′_, $I_{z_{3}}$, and *I*_1′_. These final solutions, with approximate solutions substituted for *I*_3_, $I_{z_{3}}$, and *I*_1′_, were then checked at least twice by one of the authors.

In the final solutions discussed here, all 46 coupled currents shown in Fig. 4 were expressed in terms of the “core” currents *I*_Ot_, $I_{C_{S}}$, *I*_S_, $I_{C_{B}}$, *I*_d_, *I*_c_, *I*_b_, *I*_a_, $I_{C_{A}}$, and *I*_D_. These “core” currents were then successively substituted into the remaining equations to obtain an equation for each current that was expressed as functions of *I*_Ot_. This process involved 263 algebraic substitutions of variables.

It is not feasible to list the final solutions here. Therefore, just as in the single-series “offset” case, we: (1) present numerical examples of the currents and quantum Hall voltage in Sec. 7.2; (2) list the approximate equations for the currents and quantum Hall voltage in Sec. 7.3; and (3) can supply the reader with a 29 page computer printout which lists the 263 algebraic substitutions, the exact current and quantum Hall voltage equations, the approximate equations, and numerical calculations for a particular set of circuit component values of the reader’s choice.

### 7.2 Numerical Examples

We give here numerical examples of how the parasitic impedances within the ac QHRS affect the currents and the measured values of $V_{\text{Pt}}=V_{H}(Y,Z)-r_{\text{Pt}}I_{C_{\text{Pt}}}$. Cardinal numbers are again usually assigned to the circuit element components of Fig. 4 to emphasize that the results presented below are not intended as calculations for a specific sample probe. We will measure the actual values of all the parasitic impedance components of our sample probes as a function of liquid helium level.

The cardinal values we use in most examples are

$$R_{H}=10^{4}\;\Omega$$ (38a)

$$r_{S}=r_{1}=r_{2}=r_{3}=r_{4}=r_{5}=r_{6}=r_{D}=1\;\Omega$$ (38b)

$$r_{\text{Ot}}=r_{\text{Dt}}=r_{\text{Pt}}=r_{\text{Dr}}=10^{-3}\;\Omega$$ (38c)

$$r_{a}=r_{b}=r_{c}=r_{d}=10^{-3}\;\Omega$$ (38d)

$$r_{K_{A}}=r_{K_{B}}=10^{12}\;\Omega$$ (38e)

$$C_{S}=C_{1}=C_{2}=C_{3}=C_{4}=C_{5}=C_{6}=C_{D}=10^{-10}\;F$$ (38f)

$$C_{\text{Ot}}=C_{\text{Dt}}=C_{\text{Pt}}=C_{\text{Dr}}=10^{-12}\;F$$ (38g)

$$C_{A}=C_{B}=10^{-12}\;F$$ (38h)

$$C_{1^{\prime }2^{\prime }}=C_{3^{\prime }4^{\prime }}=C_{5^{\prime }6^{\prime }}=C_{S^{\prime }D^{\prime }}=C_{S^{\prime }1^{\prime }}=C_{S^{\prime }3^{\prime }}=C_{S^{\prime }5^{\prime }}=C_{2^{\prime }D^{\prime }}=C_{4^{\prime }D^{\prime }}=C_{6^{\prime }D^{\prime }}=10^{-13}\;F$$ (38i)

$$L_{S}=L_{1}=L_{2}=L_{3}=L_{4}=L_{5}=L_{6}=L_{D}=10^{-6}\;H$$ (38j)

$$\omega =10^{4}\;\text{rad/s}.$$ (38k)

The 100 pF capacitances-to-shield values of Eq. (38f) may be close to those that will be obtained in the short NIST sample probe, but typical ac probes have values around 250 pF. Note that *C*_S′5′_ > *C*_S′3′_ > *C*_S′1′_ > *C*_S′D′_ and *C*_2′D′_ > *C*_4′D′_ > *C*_6′D′_ in real sample probes because of increasing physical separations between corresponding pairs of inner conducting surfaces.

Using these cardinal numbers, and grouping similar types of currents, the numerical results for the currents shown in Fig. 4 are

$$I_{r_{\text{Ot}}}=\{[1.00000]-j[1.0\times 10^{-11}]\}I_{\text{Ot}}$$ (39a)

$$I_{S}=\{[0.99990]-j[6.1\times 10^{-5}]\}I_{\text{Ot}}$$ (39b)

$$I_{d}=\{[0.99990]-j[1.0\times 10^{-4}]\}I_{\text{Ot}}$$ (39c)

$$I_{c}=\{[1.00000]-j[1.0\times 10^{-4}]\}I_{\text{Ot}}$$ (39d)

$$I_{b}=\{[1.00000]-j[1.0\times 10^{-4}]\}I_{\text{Ot}}$$ (39e)

$$I_{a}=\{[0.99990]-j[1.0\times 10^{-4}]\}I_{\text{Ot}}$$ (39f)

$$I_{D}=\{[0.99990]-j[3.9\times 10^{-5}]\}I_{\text{Ot}}$$ (39g)

$$I_{r_{\text{Dr}}}=\{[1.00000]+j[0.04020]\}I_{\text{Ot}}$$ (39h)

$$I_{\text{Dr}}=\{[1.00000]+j[0.04030]\}I_{\text{Ot}}$$ (39i)

$$I_{K_{B}}=\{[1.0\times 10^{-12}]+j[9.9\times 10^{-15}]\}I_{\text{Ot}}$$ (39j)

$$I_{K_{A}}=\{[1.0\times 10^{-8}]-j[1.0\times 10^{-12}]\}I_{\text{Ot}}$$ (39k)

$$I_{C_{B}}=\{-[9.9\times 10^{-11}]+j[1.0\times 10^{-8}]\}I_{\text{Ot}}$$ (39l)

$$I_{C_{A}}=\{[1.0\times 10^{-8}]+j[1.0\times 10^{-4}]\}I_{\text{Ot}}$$ (39m)

$$I_{C_{\text{Ot}}}=\{[1.0\times 10^{-22}]+j[1.0\times 10^{-11}]\}I_{\text{Ot}}$$ (39n)

$$I_{6}=\{[1.0\times 10^{-4}]+j[2.1\times 10^{-5}]\}I_{\text{Ot}}$$ (39o)

$$I_{4}=\{[1.1\times 10^{-7}]+j[2.0\times 10^{-5}]\}I_{\text{Ot}}$$ (39p)

$$I_{2}=\{[1.0\times 10^{-7}]+j[2.0\times 10^{-5}]\}I_{\text{Ot}}$$ (39q)

$$I_{6^{\prime }}=\{[1.0\times 10^{-4}]+j[9.9\times 10^{-7}]\}I_{\text{Ot}}$$ (39r)

$$I_{4^{\prime }}=\{[1.1\times 10^{-7}]+j[1.9\times 10^{-10}]\}I_{\text{Ot}}$$ (39s)

$$I_{2^{\prime }}=\{[1.0\times 10^{-7}]-j[1.0\times 10^{-11}]\}I_{\text{Ot}}$$ (39t)

$$I_{5^{\prime }}=\{[1.0\times 10^{-7}]-j[1.0\times 10^{-11}]\}I_{\text{Ot}}$$ (39u)

$$I_{3^{\prime }}=\{[1.1\times 10^{-7}]+j[1.9\times 10^{-10}]\}I_{\text{Ot}}$$ (39v)

$$I_{1^{\prime }}=\{[1.0\times 10^{-4}]+j[1.0\times 10^{-6}]\}I_{\text{Ot}}$$ (39w)

$$I_{z_{5}}=\{[1.0\times 10^{-7}]+j[2.0\times 10^{-5}]\}I_{\text{Ot}}$$ (39x)

$$I_{z_{3}}=\{[1.1\times 10^{-7}]+j[2.0\times 10^{-10}]\}I_{\text{Ot}}$$ (39y)

$$I_{z_{1}}=\{[1.0\times 10^{-4}]+j[2.1\times 10^{-5}]\}I_{\text{Ot}}$$ (39z)

$$I_{5}=\{[1.1\times 10^{-6}]+j[0.01002]\}I_{\text{Ot}}$$ (40a)

$$I_{3}=\{[1.1\times 10^{-6}]+j[0.01002]\}I_{\text{Ot}}$$ (40b)

$$I_{1}=\{[1.0\times 10^{-4}]+j[0.01002]\}I_{\text{Ot}}$$ (40c)

$$I_{C_{5}}=\{[1.0\times 10^{-6}]+j[0.01000]\}I_{\text{Ot}}$$ (40d)

$$I_{C_{3}}=\{[1.0\times 10^{-6}]+j[0.01000]\}I_{\text{Ot}}$$ (40e)

$$I_{C_{1}}=\{[1.0\times 10^{-6}]+j[0.01000]\}I_{\text{Ot}}$$ (40f)

$$I_{C_{D}}=\{[1.0\times 10^{-6}]+j[0.01000]\}I_{\text{Ot}}$$ (40g)

$$I_{C_{\text{Pt}}}=\{[1.0\times 10^{-8}]+j[1.0\times 10^{-4}]\}I_{\text{Ot}}$$ (40h)

$$I_{C_{\text{Dr}}}=\{[1.0\times 10^{-8}]+j[1.0\times 10^{-4}]\}I_{\text{Ot}}$$ (40i)

$$I_{C_{6^{\prime }D^{\prime }}}=\{[1.0\times 10^{-9}]+j[1.0\times 10^{-5}]\}I_{\text{Ot}}$$ (40j)

$$I_{C_{4^{\prime }D^{\prime }}}=\{[1.0\times 10^{-9}]+j[1.0\times 10^{-5}]\}I_{\text{Ot}}$$ (40k)

$$I_{C_{2^{\prime }D^{\prime }}}=\{[1.0\times 10^{-9}]+j[1.0\times 10^{-5}]\}I_{\text{Ot}}$$ (40l)

$$I_{C_{S^{\prime }5^{\prime }}}=\{[1.0\times 10^{-9}]+j[1.0\times 10^{-5}]\}I_{\text{Ot}}$$ (40m)

$$I_{C_{S^{\prime }3^{\prime }}}=\{[1.0\times 10^{-9}]+j[1.0\times 10^{-5}]\}I_{\text{Ot}}$$ (40n)

$$I_{C_{S^{\prime }1^{\prime }}}=\{[1.0\times 10^{-9}]+j[1.0\times 10^{-5}]\}I_{\text{Ot}}$$ (40o)

$$I_{C_{S^{\prime }D^{\prime }}}=\{[1.0\times 10^{-9}]+j[1.0\times 10^{-5}]\}I_{\text{Ot}}$$ (40p)

$$I_{C_{5^{\prime }6^{\prime }}}=\{[1.0\times 10^{-9}]+j[1.0\times 10^{-5}]\}I_{\text{Ot}}$$ (40q)

$$I_{C_{3^{\prime }4^{\prime }}}=\{[1.0\times 10^{-9}]+j[1.0\times 10^{-5}]\}I_{\text{Ot}}$$ (40r)

$$I_{C_{1^{\prime }2^{\prime }}}=\{[1.0\times 10^{-9}]+j[1.0\times 10^{-5}]\}I_{\text{Ot}}$$ (40s)

We can see from the 90° out-of-phase (imaginary or j) components of currents $I_{z_{5}}$, *I*_5′_, $I_{z_{3}}$, *I*_3′_, $I_{z_{1}}$, *I*_1′_, and *I_D_* that the four shunt currents $I_{C_{5}}$, $I_{C_{3}}$, $I_{C_{1}}$, and $I_{C_{D}}$ all bypass the QHE device. This is a great advantage of the quadruple-series circuit because it reduces the errors due to the parasitic capacitances *C*_5_, *C*_3_, *C*_1_, and *C*_D_.

The out-of-phase components of shunt currents $I_{C_{5}}$, $I_{C_{3}}$, $I_{C_{1}}$, $I_{C_{D}}$, $I_{C_{A}}$, $I_{C_{\text{pt}}}$, and $I_{C_{\text{Dr}}}$are much larger than for shunt currents $I_{C_{B}}$and $I_{C_{\text{Ot}}}$because contact pads 5*′*, 3*′*, 1*′*, and D*′* are all near the quantum Hall potential, rather than near the shield potential. A 1 % out-of-phase current passes through each of the coaxial cable capacitances *C*_5_, *C*_3_, *C*_1_, and *C*_D_in this example. That is not a problem if there is enough adjustment in the bridge Drive to provide this extra 4 % of out-of-phase current to *I*_Dr_.

The exact equation for the measured ac quantum Hall voltage is obtained by summing the voltages between the inner conductors of the Detector coaxial port and the Potential coaxial port. Taking the path from the Detector port to point Z, through arm 4, voltage generators *V*_c4_ and *V*_c3_, through arm 3 to point Y, then to the Potential port we find that

$$V_{\text{Pt}}=R_{H}I_{c}+R_{H}I_{4^{\prime }}+z_{4}I_{4}+z_{3}I_{z_{3}}-r_{\text{Pt}}I_{C_{\text{Pt}}},$$ (41)

which can be expressed as

$$V_{\text{Pt}}=V_{H}(Y,Z)-r_{\text{Pt}}I_{C_{\text{Pt}}}$$ (42a)

or

$$V_{H}(Y,Z)=[1+\Delta_{\text{YZ}}]R_{H}(i)I_{\text{Ot}},$$ (42b)

where Δ_YZ_is the correction factor to the intrinsic quantized Hall resistance *R*_H_*(i)* of the QHE device.

The numerical results for this example are

$$V_{H}(Y,Z)=\{1-[2.1\times 10^{-7}]-j[1.0\times 10^{-4}]\}R_{H}I_{\text{Ot}}$$ (43a)

$$\Delta_{\text{YZ}}=\{-[2.1\times 10^{-7}]-j[1.0\times 10^{-4}]\}$$ (43b)

for 100 pF lead capacitances and the *same* values

$$\Delta_{\text{YZ}}=\{-[2.1\times 10^{-7}]-j[1.0\times 10^{-4}]\}$$ (44)

for 250 pF coaxial leads. Therefore, the Δ_YZ_corrections are independent of the values of the capacitances-to-shield.

If the wire-to-wire capacitances can all be reduced to 1 × 10^−14^ F, then the in-phase and out-of-phase corrections are

$$\Delta_{\text{YZ}}=\{-[2.0\times 10^{-7}]-j[1.0\times 10^{-5}]\}$$ (45a)

and

$$\Delta_{\text{YZ}}=\{-[2.0\times 10^{-7}]-j[1.0\times 10^{-5}]\}$$ (45b)

for 100 pF and 250 pF coaxial leads, respectively.

There is a −1 × 10^−4^ *R*_H_*I*_Ot_ out-of-phase component in the *V*_H_(Y,Z) signal for the numerical examples given in Eqs. (43) and (44). We will see from the approximate solution listed below in Eq. (55) that these out-of-phase errors arise from the parasitic impedances.

The out-of-phase component of the ac quantum Hall voltage signal is nulled with bridge balances. Balances in NIST high precision ac bridges are capable of providing out-of-phase adjustment signals as large as 5 × 10^−4^ *R*_H_*I*_Ot_, so the bridges can null this signal. However, great care must be taken to calibrate and apply corrections for the inadvertent injection of in-phase signal resulting from the in-phase (phase defect) contributions of the capacitor components used in the bridge balances to null the out-of-phase component of the ac quantum Hall voltage signal. These inadvertent in-phase (phase defect) injection signals are unintentionally *added* to the real in-phase component of the ac quantum Hall voltage signal. The uncorrected phase defect error in the measured quantum Hall voltage has a *linear* frequency dependence in NIST four-terminal-pair bridges. (Note that normal ac reference resistors have very small capacitances and inductances, and therefore these phase defect errors can be neglected, but this is not the case for ac quantum Hall standards.) Failure to properly account for the phase defect errors may be a source of the linear frequency dependences of the ac quantized Hall resistances reported by other laboratories.

We see from Eqs. (42a), (43), (44), and (45) that the real parts of the measured ac quantum Hall voltage *V*_Pt_ and the internal voltage *V*_H_(Y,Z) appear to have unacceptably large error terms, with values of −2 × 10^−7^*R*_H_*(i)I*_Ot_ for these two examples. However, we found in Refs. [13] and [16] (and find again here) that *V*_Pt_and *V*_H_(Y,Z) are functions of the longitudinal voltage *V_x_*(2,6) along the device, where *V_x_*(2,6) is measured between room temperature access points 2 and 6. Therefore, the equation for *V*_H_(Y,Z) can be expressed two different ways: the way we have been using

$$V_{\text{Pt}}+r_{\text{Pt}}I_{C_{\text{Pt}}}=V_{H}(Y,Z)=[1+\Delta_{\text{YZ}}]R_{H}I_{\text{Ot}},$$ (46)

and

$$V_{\text{Pt}}+r_{\text{Pt}}I_{C_{\text{Pt}}}=V_{H}(Y,Z)=[1+\delta_{\text{YZ}}]R_{H}I_{\text{Ot}}-V_{x}(2,6).$$ (47)

The first term on the right hand side of Eq. (47) is the ac quantum Hall voltage that we want to extract. This term has a correction factor δ_YZ_arising from parasitic impedances in the ac quantized Hall resistance standard. The second term on the right hand side of Eq. (47) is the ac longitudinal voltage. Thus *V*_H_(Y,Z) is the ac quantum Hall voltage across the QHE device (with corrections) *minus* the ac longitudinal voltage *V_x_*(2,6) along the device.

It is crucial in Eq. (47) that *both V*_Pt_and *V_x_*(2,6) be measured under the *same* conditions. Unlike the single-series “offset” case shown in Fig. 3, no currents pass through capacitors *C*_2_ and *C*_4_ in Fig. 4. Thus, to a high degree of accuracy, there are *no* significant parasitic impedance corrections to the ac value of *V_x_*(2,6) in Eq. (47), so

$$V_{x}(2,6)≅(r_{b}+r_{c})I_{\text{Ot}}.$$ (48)

Another consequence of the requirement that both *V*_Pt_and *V_x_*(2,6) be measured under the same conditions is that *V*_Pt_and *V_x_*(2,6) should be measured during the *same* cool-down.

We suspect that the intrinsic longitudinal resistances *r*_b_and *r*_c_are not frequency dependent, and therefore that dc measurements of *V_x_*(2,6) will suffice, but the ac measurements described in Sec. 6.4 should also be made in order to test this supposition. The single-series “offset” ac *V_x_*(2,6) measurements discussed in Sec. 6.4 will have to be corrected by the factor Δ_26_ of Eq. (26) to obtain the ac values of *r*_b_and *r*_c_to be used in Eq. (48).

It follows from Eqs. (46), (47), and (48) that the correction factor δ_YZ_to the ac quantum Hall voltage *R*_H_*(i)I*_Ot_ is

$$\delta_{\text{YZ}}=\Delta_{\text{YZ}}+\frac{\left(r_{b}+r_{c}\right)}{R_{H}}.$$ (49)

The predicted values of the intrinsic ac quantum Hall voltage *R*_H_*(i)I*_Ot_ have the following correction factors δ_H_ in our numerical examples when the circuit element components shown in Fig. 4 once again have values given by Eq. (38), and the value of (*r*_b_ + *r*_c_) is 2 × 10^−7^ *R*_H_:

$$\delta_{\text{YZ}}=\{-[1.0\times 10^{-8}]-j[1.0\times 10^{-4}]\}$$ (50a)

for 100 pF lead capacitances and

$$\delta_{\text{YZ}}=\{-[1.0\times 10^{-8}]-j[1.0\times 10^{-4}]\}$$ (50b)

for 250 pF coaxial leads when the wire-to-wire capacitances are all 1 × 10^−14^ F.

If the wire-to-wire capacitances can each be reduced to 1 × 10^−13^ F, then the in-phase and out-of-phase corrections are

$$\delta_{\text{YZ}}=\{-[1.9\times 10^{-10}]-j[1.0\times 10^{-5}]\}$$ (51a)

and

$$\delta_{\text{YZ}}=\{-[1.9\times 10^{-10}]-j[1.0\times 10^{-5}]\}$$ (51b)

for 100 pF and 250 pF coaxial leads, respectively. The δ_YZ_corrections are independent of the values of the capacitances-to-shield. We will see in the approximate solution Eq. (55) that the out-of-phase and the in-phase components of Δ_YZ_ and δ_YZ_depend on the summed-total of the wire-to-wire capacitance values, and on the square of these summed-totals, respectively.

The −1 × 10^−8^ *R*_H_*I*_Ot_ error in the in-phase component of the ac quantum Hall voltage *R*_H_*(i)I*_Ot_ in Eq. (50) is quite manageable. Furthermore, it can be corrected using numerical calculations. Uncertainties can be assigned to this correction by varying the values of the circuit element components within the measurement uncertainties in the calculations, and by using the approximate solution given in Eq. (55). We have already discussed the fact that the −1 × 10^−4^ *R*_H_*I*_Ot_ out-of-phase component of *R*_H_*(i)I*_Ot_ in Eq. (50) can be handled if care is taken to calibrate and apply corrections for the small in-phase (phase defect) contributions of the bridge components used in the bridge balances to null the out-of-phase component of the ac quantum Hall voltage signal.

The quadruple-series circuit is an excellent candidate as an ac QHRS. We next give the approximate solutions for the currents and the quantum Hall voltage to show the sources of the in-phase and out-of-phase errors.

### 7.3 Approximate Quadruple-Series Solutions

The terms in the following approximate solutions were again obtained in a tedious process by changing the individual values of circuit element components shown in Fig. 4 by an order of magnitude in the computer program; observing the calculated results; and then using “educated guesses” and “trial-and-error” to find algebraic expressions that duplicate these results. The approximate solutions yield results that agree to within at least two significant figures for both the real and imaginary parts of the exact numerical results listed in Eqs. (39) to (40), (43) to (45), and (50) to (51).

All the terms in the approximate equations were found to be necessary for the particular sets of circuit component values tried so far, but other terms may very well need to be added to these approximate equations if the circuit components have values significantly different from the cardinal numbers listed in Eq. (38). The reader should again be cautioned that it is the *exact* equations that are the most reliable, not the approximate equations. However, the approximate solutions do provide physical insight into the sources of error in quantum Hall voltage measurements.

Once again, let *C*_X′X′_ be the summed-total of all the wire-to-wire capacitances,

$$\begin{array}{c} C_{X^{\prime }X^{\prime }}=C_{S^{\prime }D^{\prime }}+C_{S^{\prime }1^{\prime }}+C_{S^{\prime }3^{\prime }}+C_{S^{\prime }5^{\prime }}+C_{2^{\prime }D^{\prime }}+C_{4^{\prime }D^{\prime }}+C_{6^{\prime }D^{\prime }}\\ +C_{1^{\prime }2^{\prime }}+C_{3^{\prime }4^{\prime }}+C_{5^{\prime }6^{\prime }}. \end{array}$$ (52)

Then

$$I_{C_{\text{Ot}}}\approx I_{C_{{\text{Ot}}_{a}}}=\{[\omega^{2}C_{\text{Ot}}C_{\text{Ot}}r_{\text{Ot}}r_{\text{Ot}}]+j[\omega C_{\text{Ot}}r_{\text{Ot}}]\}I_{\text{Ot}}$$ (53a)

$$I_{r_{Ot}}\approx I_{r_{Ot_{a}}}=I_{\text{Ot}}-I_{C_{{\text{Ot}}_{a}}}$$ (53b)

$$\begin{array}{c} I_{S}\approx I_{S_{a}}=\{\left[\frac{1-r_{S}}{R_{H}}\right]-j\left[\omega C_{\text{Ot}}r_{\text{Ot}}+\omega \frac{L_{S}}{R_{H}}\right]\\ -j\omega \left(C_{2^{\prime }D^{\prime }}+C_{4^{\prime }D^{\prime }}+C_{6^{\prime }D^{\prime }}+C_{1^{\prime }2^{\prime }}+C_{3^{\prime }4^{\prime }}+C_{5^{\prime }6^{\prime }}\right)R_{H}]\}I_{\text{Ot}} \end{array}$$ (53c)

$$\begin{array}{c} I_{C_{B}}\approx I_{C_{B_{a}}}=\{\left[\omega^{2}C_{B}C_{\text{Ot}}r_{S}r_{\text{Ot}}-\omega^{2}C_{B}L_{S}\right]\\ +\left[\omega^{2}C_{B}\left(C_{2^{\prime }D^{\prime }}+C_{4^{\prime }D^{\prime }}+C_{6^{\prime }D^{\prime }}+C_{1^{\prime }2^{\prime }}+C_{3^{\prime }4^{\prime }}+C_{5^{\prime }6^{\prime }}\right)R_{H}r_{S}\right]\\ +j\left[\omega C_{B}r_{S}\right]\}I_{\text{Ot}} \end{array}$$ (53d)

$$\begin{array}{c} I_{K_{B}}\approx I_{K_{B_{a}}}=\{\left[\frac{r_{S}}{r_{K_{B}}}\right]+j\left[\omega \frac{L_{S}}{r_{K_{B}}}\right]\\ -j\left[\omega (C_{2^{\prime }D^{\prime }}+C_{4^{\prime }D^{\prime }}+C_{6^{\prime }D^{\prime }}+C_{1^{\prime }2^{\prime }}+C_{3^{\prime }4^{\prime }}+C_{5^{\prime }6^{\prime }})R_{H}\frac{r_{S}}{r_{K_{B}}}\right]\}I_{\text{Ot}} \end{array}$$ (53e)

$$I_{d}\approx I_{d_{a}}=I_{S_{a}}+I_{C_{B_{a}}}-\left\{j\left[\omega \left(C_{S^{\prime }D^{\prime }}+C_{S^{\prime }1^{\prime }}+C_{S^{\prime }3^{\prime }}+C_{S^{\prime }5^{\prime }}\right)R_{H}\right]\right\}I_{\text{Ot}}$$ (53f)

$$\begin{array}{c} I_{6}\approx I_{6_{a}}=\{\left[\frac{r_{S}}{R_{H}}\right]+j\left[\omega \frac{L_{S}}{R_{H}}+\omega C_{B}r_{S}\frac{r_{d}}{R_{H}}\right]\\ +j\left[\omega \left(C_{6^{\prime }D^{\prime }}+C_{5^{\prime }6^{\prime }}\right)R_{H}\right]\}I_{\text{Ot}} \end{array}$$ (53g)

$$\begin{array}{c} I_{C_{6^{\prime }D^{\prime }}}\approx I_{C_{6^{\prime }D_{a}^{\prime }}}=\{\left[\omega^{2}C_{X^{\prime }X^{\prime }}C_{6^{\prime }D^{\prime }}R_{H}R_{H}+\omega^{2}C_{6^{\prime }D^{\prime }}L_{D}\right]\\ +j\left[\omega C_{6^{\prime }D^{\prime }}R_{H}\right]\}I_{\text{Ot}} \end{array}$$ (53h)

$$I_{C_{5^{\prime }6^{\prime }}}\approx I_{C_{5^{\prime }6_{a}^{\prime }}}=\left\{\left[\omega^{2}C_{5^{\prime }6^{\prime }}C_{X^{\prime }X^{\prime }}R_{H}R_{H}\right]+j\left[\omega C_{5^{\prime }6^{\prime }}R_{H}\right]\right\}I_{\text{Ot}}$$ (53i)

$$\begin{array}{l} I_{6^{\prime }}\approx I_{6_{a}^{\prime }}=\{\left[\frac{r_{S}}{R_{H}}\right]+j\left[\omega \frac{L_{S}}{R_{H}}+\omega C_{B}r_{S}\frac{r_{d}}{R_{H}}\right]\\ -j\left[\omega \left(C_{X^{\prime }X^{\prime }}-C_{6^{\prime }D^{\prime }}-C_{5^{\prime }6^{\prime }}\right)r_{S}\right]\}I_{\text{Ot}} \end{array}$$ (53j)

$$\begin{array}{c} I_{5^{\prime }}\approx I_{5_{a}^{\prime }}=\{\left[\frac{r_{c}}{R_{H}}\right]+j\left[\omega C_{B}r_{S}\frac{r_{c}}{R_{H}}\right]\\ -j\left[\omega C_{X^{\prime }X^{\prime }}r_{c}\right]\}I_{\text{Ot}} \end{array}$$ (53k)

$$\begin{array}{c} I_{C_{S^{\prime }5^{\prime }}}\approx I_{C_{S^{\prime }5_{a}^{\prime }}}=\{[\omega^{2}C_{S^{\prime }5^{\prime }}C_{X^{\prime }X^{\prime }}R_{H}R_{H}-\omega^{2}C_{B}C_{S^{\prime }5^{\prime }}R_{H}r_{S}]\\ +[\omega^{2}C_{S^{\prime }5^{\prime }}L_{S}]+j[\omega C_{S^{\prime }5^{\prime }}R_{H}]\}I_{\text{Ot}} \end{array}$$ (53l)

$$\begin{array}{c} I_{z_{5}}\approx I_{z_{5_{a}}}=I_{5_{a}^{\prime }}+\{[\omega^{2}C_{X^{\prime }X^{\prime }}(C_{S^{\prime }5^{\prime }}+C_{5^{\prime }6^{\prime }})R_{H}R_{H}]\\ +j[\omega (C_{S^{\prime }5^{\prime }}+C_{5^{\prime }6^{\prime }})R_{H}]\}I_{\text{Ot}} \end{array}$$ (53m)

$$\begin{array}{c} I_{C_{5}}\approx I_{C_{5_{a}}}=\{[\omega^{2}C_{5}C_{X^{\prime }X^{\prime }}R_{H}R_{H}-\omega^{2}C_{B}C_{5}R_{H}r_{S}]\\ +j[\omega C_{5}R_{H}]\}I_{\text{Ot}} \end{array}$$ (53n)

$$I_{5}\approx I_{5_{a}}=I_{z_{5_{a}}}+I_{C_{5_{a}}}$$ (53o)

$$I_{c}\approx I_{c_{a}}=I_{d_{a}}-I_{5_{a}^{\prime }}+I_{6_{a}^{\prime }}$$ (53p)

$$\begin{array}{c} I_{4}\approx I_{4_{a}}=\{\left[\frac{r_{c}}{R_{H}}+\frac{r_{S}r_{6}}{R_{H}R_{H}}\right]+j\left[\omega C_{B}r_{S}\frac{r_{b}}{R_{H}}\right]\\ +j\left[\omega \frac{L_{S}}{R_{H}}\frac{r_{6}}{R_{H}}+\omega \frac{L_{6}}{R_{H}}\frac{r_{S}}{R_{H}}\right]\\ +j[\omega (C_{4^{\prime }D^{\prime }}+C_{3^{\prime }4^{\prime }})R_{H}]\}I_{\text{Ot}} \end{array}$$ (53q)

$$\begin{array}{c} I_{C_{4^{\prime }D^{\prime }}}\approx I_{C_{4^{\prime }D_{a}^{\prime }}}=\{[\omega^{2}C_{X^{\prime }X^{\prime }}C_{4^{\prime }D^{\prime }}R_{H}R_{H}+\omega^{2}C_{4^{\prime }D^{\prime }}L_{D}]\\ +j[\omega C_{4^{\prime }D^{\prime }}R_{H}]\}I_{\text{Ot}} \end{array}$$ (53r)

IC3′4′≈IC3′4′a={[ω2CX′X′C3′4′RHRH]+j[ωC3′4′RH]}IOt (53s)

$$I_{4^{\prime }}\approx I_{4_{a}^{\prime }}=I_{4_{a}}-\{j[\omega (C_{4^{\prime }D^{\prime }}+C_{3^{\prime }4^{\prime }})R_{H}+\omega C_{X^{\prime }X^{\prime }}r_{c}]\}I_{\text{Ot}}$$ (53t)

$$\begin{array}{c} I_{3^{\prime }}\approx I_{3_{a}^{\prime }}=\{\left[\frac{r_{b}}{R_{H}}+\frac{r_{1}r_{D}}{R_{H}R_{H}}\right]+j\left[\omega C_{B}r_{S}\frac{r_{c}}{R_{H}}\right]\\ +j\left[\omega \frac{L_{1}}{R_{H}}\frac{r_{D}}{R_{H}}+\omega \frac{L_{D}}{R_{H}}\frac{r_{1}}{R_{H}}\right]\\ -j\left[\omega C_{X^{\prime }X^{\prime }}r_{c}\right]\}I_{\text{Ot}} \end{array}$$ (53u)

$$\begin{array}{l} I_{C_{S^{\prime }3^{\prime }}}\approx I_{C_{S^{\prime }3_{a}^{\prime }}}=\{[\omega^{2}C_{S^{\prime }3^{\prime }}C_{X^{\prime }X^{\prime }}R_{H}R_{H}]\\ +[\omega^{2}C_{S^{\prime }3^{\prime }}L_{S}]+j[\omega C_{S^{\prime }3^{\prime }}R_{H}]\}I_{\text{Ot}} \end{array}$$ (53v)

$$I_{z_{3}}\approx I_{z_{3_{a}}}=I_{3_{a}^{\prime }}+\{j[\omega (C_{S^{\prime }3^{\prime }}+C_{3^{\prime }4^{\prime }})R_{H}]\}I_{\text{Ot}}$$ (53w)

$$\begin{array}{c} I_{C_{3}}\approx I_{C_{3_{a}}}=\{[\omega^{2}C_{3}C_{X^{\prime }X^{\prime }}R_{H}R_{H}-\omega^{2}C_{B}C_{3}R_{H}r_{S}]\\ +j[\omega C_{3}R_{H}]\}I_{\text{Ot}} \end{array}$$ (53x)

$$I_{3}\approx I_{3_{a}}=I_{z_{3_{a}}}+I_{C_{3_{a}}}$$ (53y)

$$I_{b}\approx I_{b_{a}}=I_{c_{a}}-I_{3_{a}^{\prime }}+I_{4_{a}^{\prime }}$$ (53z)

$$\begin{array}{l} I_{2}\approx I_{2_{a}}=\{\left[\frac{r_{b}}{R_{H}}+\omega^{2}C_{X^{\prime }X^{\prime }}\left(C_{2^{\prime }D^{\prime }}+C_{1^{\prime }2^{\prime }}\right)R_{H}R_{H}\right]\\ +j\left[\omega C_{B}r_{S}\frac{r_{b}}{R_{H}}+\omega (C_{2^{\prime }D^{\prime }}+C_{1^{\prime }2^{\prime }})R_{H}\right]\}I_{\text{Ot}} \end{array}$$ (54a)

$$\begin{array}{c} I_{C_{2^{\prime }D^{\prime }}}\approx I_{C_{2^{\prime }D_{a}^{\prime }}}=\{[\omega^{2}C_{X^{\prime }X^{\prime }}C_{2^{\prime }D^{\prime }}R_{H}R_{H}+\omega^{2}C_{2^{\prime }D^{\prime }}L_{D}]\\ +j[\omega C_{2^{\prime }D^{\prime }}R_{H}]\}I_{\text{Ot}} \end{array}$$ (54b)

$$I_{C_{1^{\prime }2^{\prime }}}\approx I_{C_{1^{\prime }2_{a}^{\prime }}}=\{[\omega^{2}C_{X^{\prime }X^{\prime }}C_{1^{\prime }2^{\prime }}R_{H}R_{H}]+j[\omega C_{1^{\prime }2^{\prime }}R_{H}]\}I_{\text{Ot}}$$ (54c)

$$\begin{array}{l} I_{2^{\prime }}\approx I_{{2^{\prime }}_{a}}=I_{2_{a}}-\{[\omega^{2}C_{X^{\prime }X^{\prime }}(C_{2^{\prime }D^{\prime }}+C_{1^{\prime }2^{\prime }})R_{H}R_{H}]\\ -j[\omega (C_{2^{\prime }D^{\prime }}+C_{1^{\prime }2^{\prime }})R_{H}+\omega C_{X^{\prime }X^{\prime }}r_{c}]\}I_{\text{Ot}} \end{array}$$ (54d)

$$\begin{array}{l} I_{1^{\prime }}\approx I_{1_{a}^{\prime }}=\{\left[\frac{r_{D}}{R_{H}}\right]+j\left[\omega C_{B}r_{S}\frac{r_{D}}{R_{H}}+\omega C_{A}r_{D}+\omega \frac{L_{D}}{R_{H}}\right]\\ -j\left[\omega \left(C_{X^{\prime }X^{\prime }}-C_{S^{\prime }1^{\prime }}-C_{1^{\prime }2^{\prime }}\right)r_{D}+\omega C_{X^{\prime }X^{\prime }}r_{c}\right]\}I_{\text{Ot}} \end{array}$$ (54e)

$$\begin{array}{l} I_{C_{S^{\prime }1^{\prime }}}\approx I_{C_{S^{\prime }1_{a}^{\prime }}}=\{[\omega^{2}C_{S^{\prime }1^{\prime }}C_{X^{\prime }X^{\prime }}R_{H}R_{H}]\\ +[\omega^{2}C_{S^{\prime }1^{\prime }}L_{S}]+j[\omega C_{S^{\prime }1^{\prime }}R_{H}]\}I_{\text{Ot}} \end{array}$$ (54f)

$$I_{z_{1}}\approx I_{z_{1_{a}}}=I_{1_{a}^{\prime }}+\{j[\omega (C_{S^{\prime }1^{\prime }}+C_{1^{\prime }2^{\prime }})R_{H}]I_{\text{Ot}}$$ (54g)

$$\begin{array}{c} I_{C_{1}}\approx I_{C_{1_{a}}}=\{[\omega^{2}C_{1}C_{X^{\prime }X^{\prime }}R_{H}R_{H}-\omega^{2}C_{B}C_{1}R_{H}r_{S}]\\ +j[\omega C_{1}R_{H}]I_{\text{Ot}} \end{array}$$ (54h)

$$I_{1}\approx I_{1_{a}}=I_{z_{1_{a}}}+I_{C_{1_{a}}}$$ (54i)

Ia≈Iaa=Iba−I1′a+I2′a (54j)

$$\begin{array}{c} I_{C_{S^{\prime }D^{\prime }}}\approx I_{C_{{S^{\prime }D^{\prime }}_{a}}}=\{[\omega^{2}C_{S^{\prime }D^{\prime }}C_{X^{\prime }X^{\prime }}R_{H}R_{H}]\\ +[\omega^{2}C_{S^{\prime }D^{\prime }}(L_{S}+L_{D})]+j[\omega C_{S^{\prime }D^{\prime }}R_{H}]\}I_{\text{Ot}} \end{array}$$ (54k)

$$I_{K_{A}}\approx I_{K_{A_{a}}}=\left\{\left[\frac{R_{H}}{r_{K_{A}}}\right]-j\left[\omega C_{B}r_{S}\frac{R_{H}}{r_{K_{A}}}\right]\right\}I_{\text{Ot}}$$ (54l)

$$\begin{array}{l} I_{C_{A}}\approx I_{C_{A_{a}}}=\{[\omega^{2}C_{A}C_{X^{\prime }X^{\prime }}R_{H}R_{H}]\\ +[\omega^{2}C_{A}L_{D}]+j[\omega C_{A}R_{H}]\}I_{\text{Ot}} \end{array}$$ (54m)

$$\begin{array}{c} I_{D}\approx I_{D_{a}}=\{\left[1-\frac{r_{D}}{R_{H}}\right]+j\left[\omega C_{A}R_{H}-\omega \frac{L_{D}}{R_{H}}\right]\\ -j\left[\omega \left(C_{S^{\prime }1^{\prime }}+C_{S^{\prime }3^{\prime }}+C_{S^{\prime }5^{\prime }}+C_{1^{\prime }2^{\prime }}+C_{3^{\prime }4^{\prime }}+C_{5^{\prime }6^{\prime }}\right)R_{H}\right]\}I_{\text{Ot}} \end{array}$$ (54n)

$$\begin{array}{c} I_{C_{D}}\approx I_{C_{D_{a}}}=\{[\omega^{2}C_{D}C_{X^{\prime }X^{\prime }}R_{H}R_{H}]\\ -[\omega^{2}C_{B}C_{D}R_{H}r_{S}]+j[\omega C_{D}R_{H}]\}I_{\text{Ot}} \end{array}$$ (54o)

$$\begin{array}{c} I_{C_{\text{Pt}}}\approx I_{C_{{\text{Pt}}_{a}}}=\{[\omega^{2}C_{\text{Pt}}C_{X^{\prime }X^{\prime }}R_{H}R_{H}]\\ -[\omega^{2}C_{B}C_{\text{Pt}}R_{H}r_{S}]+j[\omega C_{\text{Pt}}R_{H}]\}I_{\text{Ot}} \end{array}$$ (54p)

$$I_{r_{\text{Dr}}}\approx I_{r_{{\text{Dr}}_{a}}}=I_{\text{Ot}}+I_{C_{5_{a}}}+I_{C_{3_{a}}}+I_{C_{1_{a}}}+I_{C_{A_{a}}}+I_{C_{D_{a}}}$$ (54q)

$$\begin{array}{c} I_{C_{\text{Dr}}}\approx I_{C_{{\text{Dr}}_{a}}}=\{[\omega^{2}C_{Dr}C_{X^{\prime }X^{\prime }}R_{H}R_{H}]\\ -[\omega^{2}C_{B}C_{\text{Dr}}R_{H}r_{S}]+j[\omega C_{\text{Dr}}R_{H}]\}I_{\text{Ot}} \end{array}$$ (54r)

$$I_{\text{Dr}}\approx I_{{\text{Dr}}_{a}}=I_{\text{Ot}}+I_{C_{5_{a}}}+I_{C_{3_{a}}}+I_{C_{1_{a}}}+I_{C_{A_{a}}}+I_{C_{D_{a}}}.$$ (54s)

Expressing Eq. (41) in the form (42),

$$\begin{array}{c} \Delta_{\text{YZ}}\approx \Delta_{{\text{YZ}}_{a}}=\{-\left[\frac{\left(r_{b}+r_{c}\right)}{R_{H}}-\frac{\left(r_{2}r_{b}+r_{5}r_{c}\right)}{R_{H}R_{H}}\right]\\ -[\omega^{2}C_{X^{\prime }X^{\prime }}C_{X^{\prime }X^{\prime }}R_{H}R_{H}-\omega^{2}C_{B}C_{X^{\prime }X^{\prime }}R_{H}r_{S}]\\ -[\omega^{2}C_{X^{\prime }X^{\prime }}L_{S}+\omega^{2}C_{B}L_{S}]\\ -j[\omega C_{X^{\prime }X^{\prime }}R_{H}-\omega C_{B}r_{S}+\omega C_{\text{Ot}}r_{\text{Ot}}+\omega C_{\text{Pt}}r_{\text{Pt}}]\}. \end{array}$$ (55)

It would have been difficult to predict some of these approximate current and voltage solutions without first knowing the exact results.

We see from Eq. (55) that the main source of the in-phase and out-of-phase corrections to the quantum Hall voltage signal arises from terms involving the summed-total, *C*_X′X′_, of the wire-to-wire capacitances. It is therefore important to minimize these wire-to-wire capacitances in ac quantized Hall resistance sample probes.

## 8. Conclusions

We have used an equivalent electrical circuit model of the quantum Hall effect device to calculate the effects of parasitic impedances that are present in four-terminal-pair [23,24] measurements of ac quantized Hall resistance standards. The discrete circuit components include the minimum number of externally measurable parasitic capacitances, inductances, and leakage resistances necessary to account for the electrical characteristics of the standard.

Both exact and approximate algebraic equations have been derived for the currents and quantum Hall voltages for single-series “offset” and quadruple-series circuit connections to the device. We predict that the quadruple-series connections are the *only* ones that can meet our desired goal of measuring the quantized Hall resistance *R*_H_ with an absolute accuracy of 10^−8^ *R*_H_ or better in the *same* cool-down for *both* ac and dc currents with *all* sample probe leads attached. We also predict that the single-series “offset” connections can be used to adequately measure the ac longitudinal resistance *R_x_* during that same cool-down.

It is crucial in these measurements of *R*_H_ and *R_x_* that the wire-to-wire capacitances of the quantum Hall device and its sample holder be made as small as possible. It is also crucial that care be taken to calibrate and apply corrections for the in-phase (phase defect) contributions of the bridge components used in the bridge balances to null the out-of-phase component of the ac quantum Hall voltage and ac longitudinal voltage signals.

Finally, we caution the reader that this analysis applies *only* to the ac quantized Hall resistance standard itself. The analysis does *not* consider the effects of (a) improper realization of the four-terminal-pair measurement definition; (b) systematic errors in the ac and dc bridges; and (c) inadequate frequency dependence corrections of the ac reference impedance standards to which the ac quantized Hall resistance standard is compared.

## Footnotes

1 Certain commercial equipment, instruments, or materials are identified in this paper to foster understanding. Such identification does not imply recommendation or endorsement by the National Institute of Standards and Technology, nor does it imply that the materials or equipment identified are necessarily the best available for the purpose.
